# Optimization of CRISPR/Cas9‐mediated *CtPDS* knockout in guar protoplasts

**DOI:** 10.1002/tpg2.70177

**Published:** 2026-02-11

**Authors:** Protik Kumar Ghosh, Sudip Biswas, Roly Malaker, Hanh Pham, Endang M. Septiningsih, Waltram Ravelombola

**Affiliations:** ^1^ Department of Soil and Crop Sciences Texas A&M University College Station Texas USA; ^2^ Texas A&M AgriLife Research‐Vernon Vernon Texas USA; ^3^ Texas A&M AgriLife Research‐Lubbock Lubbock Texas USA

## Abstract

Guar (*Cyamopsis tetragonoloba* L. Taub.) is a climate‐resilient legume with industrial and agricultural applications. Recently, gene editing has emerged as a key genetic tool for crop improvement. Despite its recent increasing value as a commodity for various uses, there is no documented report of gene editing work in guar to date. In this study, we present the first optimized protocol for protoplast‐based clustered regularly interspaced short palindromic repeats/CRISPR‐associated protein 9 (CRISPR/Cas9) genome editing in guar. The most intact and viable protoplasts were observed in the cotyledons of 6‐day‐old seedlings that were isolated using the tape‐sandwich method. Enzymatic digestion with 1.5% cellulase RS and 0.3% pectinase in 600 mM mannitol, followed by 10 min of vacuum infiltration, increased protoplast release and viability. Polyethylene glycol (PEG)‐mediated transformation had been tailored using 40% PEG for 5 min with green fluorescent protein plasmid, and the cestrum yellow leaf curling virus promoter at room temperature showed the highest transient expression efficiency (∼57%). A multiplexed CRISPR/Cas9 construct was designed to target the *Cyamopsis tetragonoloba phytoene desaturase* (*CtPDS*) gene in guar. Polymerase chain reaction amplification and Sanger sequencing of transfected protoplasts confirmed highly efficient editing, with fragment deletions ranging from 714 to 1061 bp in *CtPDS*. Overall, we achieved 100% editing efficiency, as all successfully transformed samples showed CRISPR/Cas9‐induced mutations. These findings establish a reliable, transient protoplast system for functional genomics and targeted trait improvement in guar, providing a key foundation for future crop improvement.

## Core Ideas

- Tape–sandwich method in 6‐day‐old seedlings gave the highest protoplast yield and viability.
- Polyethylene glycol (PEG)‐mediated clustered regularly interspaced short palindromic repeats/CRISPR‐associated protein 9 (CRISPR/Cas9) delivery achieved up to 57% transformation efficiency in guar.
- Cestrum yellow leaf curling virus promoter (CmYLCV) promoter outperformed cauliflower mosaic virus 35S promoter (CaMV35S) for transient green fluorescent protein (GFP) expression in guar protoplasts.
- CRISPR/Cas9 editing of *Cyamopsis tetragonoloba phytoene desaturase* (*CtPDS*) gene yielded large deletions with high sgRNA activity.

## Plain Language Summary

Guar is a vital legume for both agriculture and industry. However, no reliable gene‐editing system has been reported for guar despite its recent increase in value for various uses. In this study, we report the first successful method for performing clustered regularly interspaced short palindromic repeats/CRISPR‐associated protein 9–based genome editing in guar protoplasts with high efficiency. Our results confirmed the effectiveness and precision of the genome editing system in guar. This advancement may facilitate more rapid and precise breeding to enhance stress tolerance, yield, and gum quality in this underutilized crop.

**Abbreviations**
BF: bright‐field
BSA: bovine serum albumin
CaMV35S: cauliflower mosaic virus 35S promoter
CmYLCV: cestrum yellow leaf curling virus promoter
CRISPR/Cas9: clustered regularly interspaced short palindromic repeats/CRISPR‐associated protein 9
*CtPDS*
: *Cyamopsis tetragonoloba phytoene desaturase*
FDA: fluorescein diacetate
FL: fluorescence
GFP: green fluorescent protein
LSD: least significant difference
NC: negative control
PCR: polymerase chain reaction
PEG: polyethylene glycol
RE: restriction enzyme
RT: room temperature
sgRNA: single‐guide RNA
WT: wild type

## 1 INTRODUCTION

Guar (*Cyamopsis tetragonoloba* L. Taub., 2*n* = 2*x* = 14) is a climate‐resilient legume recognized for its drought and heat tolerance and nitrogen‐fixing capacity. These traits make it well‐suited for dryland cropping systems in arid and semiarid regions, including the southern United States (Morris, 2010; Ravelombola et al., 2021). Its economic importance lies in guar gum, a galactomannan polysaccharide extracted from the seed endosperm, which is widely used in oil and gas, food, pharmaceutical, and textile industries (Awan et al., 2024; Manna et al., 2024; Sharahi et al., 2025; Tahmouzi et al., 2023). Although guar is agriculturally and industrially important, no prior successful gene editing efforts have been reported to date, mainly due to its recalcitrant nature (Naoumkina et al., 2008).

Recent genomic studies have improved our knowledge of guar genetic diversity and population structure, providing a valuable foundation for molecular breeding (Malani et al., 2024; Ravelombola et al., 2021). Among emerging technologies, clustered regularly interspaced short palindromic repeats/CRISPR‐associated protein 9 (CRISPR/Cas9) genome editing stands out as a precise, efficient, and versatile method for trait improvement in both diploids and polyploids (Doudna & Charpentier, 2014; Thomson et al., 2022; Wilson et al., 2019; Xie & Yang, 2013). Although targeted gene regulation has been demonstrated in guar using approaches such as *Agrobacterium*‐mediated RNA interference of the *galactomannan galactosyltransferase* gene (Verma et al., 2024), but stable genome editing through CRISPR/Cas9 has not yet been reported in this species.

To bridge this research gap, we developed a transient expression system, polyethylene glycol (PEG)‐mediated protoplast transformation, as a genetic tool to validate CRISPR/Cas9 constructs in guar. Transient assays, including protoplast transformation, facilitate the optimization of single‐guide RNA (sgRNA) activity, promoter efficiency, and construct design (Bridgeland et al., 2023; Lin et al., 2018; Shan et al., 2014; Tsakirpaloglou et al., 2023). This system has been successfully applied in legumes such as cowpea (*Vigna unguiculata* L.), peanut (*Arachis hypogaea* L.), and chickpea (*Cicer arietinum* L.) (Badhan et al., 2021; Biswas, Bridgeland, et al., 2022; Biswas, Wahl, et al., 2022; Bridgeland et al., 2023; Irum et al., 2025). However, optimization protocols have not yet been developed for guar.

Toward this aim, we targeted the *Cyamopsis tetragonoloba phytoene desaturase* (*CtPDS*) gene using a CRISPR/Cas9 construct containing three multiplexed sgRNAs. Knocking out *PDS* is expected to produce a visible bleaching phenotype, allowing straightforward visual confirmation of editing efficiency (Bridgeland et al., 2023; Nekrasov et al., 2013; Soyars et al., 2018). This study will serve as the first step toward a reproducible gene editing framework in guar, supporting future functional genomics and targeted trait improvement in this underutilized crop.

## 2 MATERIALS AND METHODS

### 2.1 Plant materials

Seeds of the guar variety Kinman (Brooks × Mills) were used in this study due to its high yield, adaptability, and disease resistance (Stafford et al., 1976). It is also useful for industrial applications, as it matures earlier than other alternatives and has a high gum content. Due to its higher seed yield and ability to adapt to the guar‐growing regions of Oklahoma and Texas, Kinman is the ideal candidate for this investigation.

### 2.2 Protoplast isolation

Protoplast isolation was performed using cotyledons, unifoliate leaves, and trifoliate leaves from 6‐,12‐, and 24‐day‐old guar seedlings. Three tissue preparation methods were tested: (a) the leaf‐cutting method, (b) the tape‐sandwich method, and (c) the strip method. Tissue preparation steps were adapted from protocols by Shan et al. (2014), Wu and Hanzawa (2018), and Biswas, Bridgeland, et al. (2022) for the leaf‐cutting method, Wu et al. (2009) and Bridgeland et al. (2023) for the tape‐sandwich method, and Irum et al. (2025) for the strip method. In the leaf‐cutting approach, cotyledons, unifoliate leaves, and trifoliate leaves were harvested, midribs were removed, and approximately 1 g of tissue per trial (3 g total) was cut into ∼0.5 mm wide latitudinal strips and placed directly into the enzyme solution. In the tape‐sandwich method, the upper epidermal surface of the leaves was affixed to Fisherbrand Colored Labeling Tape. A strip of 3 M Magic Tape (standard clear office tape) was gently applied to the lower epidermis and peeled away to remove it. The leaves, with the upper epidermis still attached to the labeling tape, were transferred to a Petri dish containing enzyme solution. In the strip method, the leaves were left intact with their midribs and latitudinal incisions were made on both sides of the midrib through the lamina without severing the midrib itself. The petiole was used to handle and immerse the entire leaf in the enzyme solution, ensuring that the internal tissues were exposed for digestion while maintaining the structural integrity of the leaf. Based on comparative evaluations, the tape‐sandwich method was selected for downstream transformation experiments due to its higher yield.

To determine the optimal enzyme mixture, preliminary optimization experiments were conducted by varying the concentrations of mannitol (serving as an osmotic stabilizer) and enzymes. Mannitol concentrations of 400, 600, and 800 mM were compared, along with cellulase concentrations of 0.75%, 1.0%, 1.5%, and 2.0% (both cellulase R‐10 and cellulase RS). Trials were also carried out with and without the addition of pectinase. From these evaluations, the combination of 600 mM mannitol, 1.5% cellulase RS, 0.4% macerozyme R‐10, and 0.3% pectinase provided the best balance of protoplast yield and viability.

The optimized enzyme solution used for downstream experiments consisted of 1.5% (w/v) cellulase RS, 0.4% (w/v) macerozyme R‐10, 0.3% (w/v) pectinase from *Aspergillus niger*, 600 mM mannitol, 20 mM KCl, 10 mM CaCl_2_, 0.1% (w/v) bovine serum albumin (BSA), and 20 mM MES (pH 5.7). Tissue samples were fully submerged in 20 mL of enzyme solution in a Petri dish at a ratio of 10 mL solution per gram of leaf tissue. The Petri dish was covered with aluminum foil and subjected to vacuum infiltration at 380–508 mmHg in the dark. Vacuum infiltration was tested for 10, 20, and 30 min, and a 10‐min duration was maintained for all subsequent experiments. After vacuum treatment, samples were incubated for 1–1.5 h at room temperature (RT; approximately 25 ± 2°C) with gentle shaking at 45 rpm using an orbital shaker (Thermo Scientific MaxQ 2000). During the initial incubation period, protoplasts were released into the enzyme solution along with cellular debris, residual enzymes, phenolic compounds, and other metabolites that can compromise protoplast viability (Kennedy & De Filippis, 2004). To minimize potential cytotoxic effects, after initial digestion and debris release, the enzyme solution was discarded. Fresh enzyme solution was then added to the leaves, and incubation continued for an additional 3 h at 45 rpm. The solution gradually turned greenish, indicating continued release of viable protoplasts.

**Core Ideas**

- Tape–sandwich method in 6‐day‐old seedlings gave the highest protoplast yield and viability.
- Polyethylene glycol (PEG)‐mediated clustered regularly interspaced short palindromic repeats/CRISPR‐associated protein 9 (CRISPR/Cas9) delivery achieved up to 57% transformation efficiency in guar.
- Cestrum yellow leaf curling virus promoter (CmYLCV) promoter outperformed cauliflower mosaic virus 35S promoter (CaMV35S) for transient green fluorescent protein (GFP) expression in guar protoplasts.
- CRISPR/Cas9 editing of *Cyamopsis tetragonoloba phytoene desaturase* (*CtPDS*) gene yielded large deletions with high sgRNA activity.

Following enzymatic digestion, the enzyme solution containing released protoplasts was poured into a 50 mL Falcon tube. An equal volume of washing solution (W5: 600 mM mannitol, 154 mM NaCl, 5 mM KCl, 125 mM CaCl_2_, and 2 mM MES, pH 5.7) was added, and the mixture was slowly agitated by hand for 10 s to obtain even suspension of protoplasts and prevent clumping, thereby maximizing yield and maintaining cell viability (Král et al., 2025). The suspension was passed through a 40‐µm nylon mesh for two to three times, and each time the filtrate was collected into a fresh 50‐mL Falcon tube. Afterwards, the filtrate was centrifuged at 100 × *g* for 3 min at RT using a Beckman Coulter Allegra X‐14R centrifuge. The supernatant was removed, and the pellet was resuspended in 10 mL of W5 solution. After a second centrifugation under the same conditions, the protoplasts were resuspended in approximately 10–12 mL of incubation solution (WS1: 600 mM mannitol, 20 mM KCl, 10 mM CaCl_2_, 0.1% (w/v) BSA, and 4 mM MES, pH 5.7) and stored at 4°C. All solutions used throughout the procedure were prepared with autoclaved water and filtered through a 0.2‐µm syringe filter before use.

### 2.3 Protoplast counting and viability test

Protoplasts were counted using a hemocytometer (Revolve‐100‐G, Echo Laboratories) under 10x magnification. The number of protoplasts in four squares of the hemocytometer grid was recorded, and protoplast concentration was calculated using the following formula given by Biswas, Wahl, et al. (2022):

$$\begin{array}{ccc} & & \mathrm{Protoplast}\;\mathrm{concentration}\;\left(\mathrm{cells}\;mL^{-1}\right)\\ & & \;=\;\mathrm{average}\;\mathrm{count}\;\mathrm{per}\;\mathrm{square}\times 10^{4} \end{array}$$

The total protoplast yield per gram of tissue was then estimated by multiplying the protoplast concentration (cells mL^−1^) by the resuspension volume (mL) and dividing by the weight of the tissue (g), according to the formula:

$$\begin{array}{ccc} & & \mathrm{Total}\;\mathrm{protoplast}\;\mathrm{yield}\;\left(\mathrm{per}\;g\;\mathrm{of}\;\mathrm{tissue}\right)\\ & & \;=\;\{\mathrm{protoplast}\;\mathrm{concentration}\;\left(\mathrm{cells}\;mL^{-1}\right)\\ & & \;\times \;\mathrm{resuspension}\;\mathrm{volume}\;\left(\mathrm{mL}\right)\}/\mathrm{tissue}\;\mathrm{weight}\;\left(g\right). \end{array}$$

Protoplast viability was assessed using fluorescein diacetate (FDA) and propidium bromide (Sigma‐Aldrich) staining, following the manufacturer's protocol. A working solution was prepared by adding 1 µL each of FDA and propidium bromide to 98 µL of nuclease‐free water. Then, 10 µL of this 10x staining solution was added to 90 µL of protoplast suspension and mixed gently by tapping. After a 2‐min incubation at RT, the viability of the protoplasts was evaluated using an Echo Revolve microscope under ultraviolet light. Viable protoplasts appeared green, while dead cells and cell debris remained unstained. The protoplast viability percentage was computed using the following formula given by Biswas, Wahl, et al. (2022):

$$\begin{array}{ccc} & & \mathrm{Viable}\;\mathrm{protoplasts}\;\left(\%\right)=(\mathrm{number}\;\mathrm{of}\;\mathrm{green}\\ & & \;-\;\mathrm{stained}\;\mathrm{protoplasts}\;\mathrm{observed}\;\mathrm{under}\;\mathrm{fluorescence}\\ & & \;/\mathrm{total}\;\mathrm{number}\;\mathrm{of}\;\mathrm{protoplasts}\;\mathrm{observed}\;\mathrm{under}\;\mathrm{bright}\\ & & \;-\;\mathrm{field})\times 100. \end{array}$$

For imaging, the same microscopic fields were captured under both bright‐field (BF) and fluorescence (FL) channels to ensure accurate correlation between cell morphology and FL signal.

### 2.4 Protoplast transformation

Before transformation, WS1‐incubated protoplasts were centrifuged again at 100 × *g* for 3 min at RT, and an equivalent volume of MMG solution (600 mM mannitol, 15 mM MgCl_2_, 0.1% [w/v] BSA, and 4 mM MES, pH 5.7) was used to resuspend the pellet. MMG solution acts as a protective and supportive medium for PEG‐mediated protoplast transformation by maintaining osmotic stability and protoplast viability, thereby creating optimal conditions for DNA uptake and membrane integrity (Yoo et al., 2007). The pTRANS_100_*CtPDS* CRISPR/Cas9 construct along with cauliflower mosaic virus 35S promoter:green fluorescent protein (CaMV35S:GFP) and cestrum yellow leaf curling virus promoter:GFP (CmYLCV:GFP) vectors were used for PEG‐mediated transformation of guar protoplasts at 13°C and RT. The CaMV35S:GFP and CmYLCV:GFP vectors served as positive controls to assess transformation efficiency in guar protoplasts. A total of 60 µg of each plasmid (pTRANS_100_*CtPDS*, CaMV35S, and CmYLCV) was added to 400 µL of guar protoplast suspension (approximately 2.5 × 10⁵ cells), and the mixture was carefully poured into a 15 mL sterile Falcon tube. Different PEG concentrations (20% and 40%) and incubation times (5 and 10 min) were tested before the final experiment. For the transformation, a 40% PEG solution (200 mM mannitol, 100 mM CaCl_2_, 40% PEG 4000) was freshly prepared, 460 µL was added to the tube, and the mixture was gently inverted. The mixture was then incubated in the dark for 5 min to facilitate plasmid DNA uptake. To terminate the transformation process and clean the PEG solution, 5 mL of WS1 solution was added and mixed gently by inversion. Finally, the suspension underwent centrifugation at 250 × *g* for 1 min at RT. PEG‐containing supernatant was carefully removed to the greatest extent possible, and the resulting pellet was resuspended in 2 mL of WS1 solution.

pTRANS_100_*CtPDS*‐transformed protoplasts were incubated in the dark at RT for at least 24 h with the tubes wrapped in aluminum foil. For CaMV35S‐ and CmYLCV‐transfected protoplasts, transformation efficiency was assessed after 14 h of incubation by counting GFP‐positive protoplasts under FL microscopy and calculating their proportion relative to the total number of protoplasts observed under BF microscopy. Transformation efficiency (%) was calculated as the average of three replicates using the following formula:

$$\begin{array}{ccc} & & \mathrm{Transformation}\;\mathrm{efficiency}\;\left(\%\right)=(\mathrm{number}\;\mathrm{of}\;\mathrm{GFP}\\ & & \;-\;\mathrm{positive}\;\mathrm{protoplasts}\;\mathrm{counted}\;\mathrm{under}\;\mathrm{FL}\\ & & \;/\mathrm{total}\;\mathrm{number}\;\mathrm{of}\;\mathrm{protoplasts}\;\mathrm{observed}\;\mathrm{under}\;\mathrm{BF})\\ & & \;\times \;100. \end{array}$$

### 2.5 *CtPDS* gene validation and sgRNA design

The DNA sequence of *CtPDS* gene was retrieved from the National Center for Biotechnology Information database (https://www.ncbi.nlm.nih.gov/) based on the genome assembly GCA_026586085.1 and used as input for sgRNA design. The genomic and transcriptomic evidence strongly supports the notion that soybean (*Glycine max* (L.) Merr.) and guar are closely related legumes with conserved gene sequences and functions (Criollo Delgado et al., 2025). This relationship was further supported by a phylogenetic analysis of *PDS* genes from multiple legumes, including chickpea, pea (*Pisum sativum* L.), soybean, adzuki bean (*Vigna angularis* (Willd.) Ohwi & Ohashi), rice bean (*Vigna umbellata* (Thunb.) Ohwi & Ohashi), mungbean (*Vigna radiata* (L.) R. Wilczek), cowpea, common bean (*Phaseolus vulgaris* L.), pigeonpea (*Cajanus cajan* (L.) Huth), peanut, and guar, constructed using Geneious Prime (version 2025.2) (www.geneious.com) (Figure S1). As the guar genome was available as a contig‐level assembly without complete gene annotation, we identified the exon structure of the *PDS* gene in soybean (13 exons, based on the highest sequence coverage). We used these as a reference to annotate the corresponding exons in the guar genome. This was achieved by performing Multiple Basic Local Alignment Search tool searches of the soybean *PDS* exons against the guar genome sequence (Table S1). To validate the identified *CtPDS* sequence and ensure accurate sgRNA targeting, a pair of primers (forward primer: 5′‐TCTGTCTCTGCAACCTTCCA‐3′, reverse primer: 5′‐GGACACAGCTAGAGAGGAACA‐3′) was designed using Primer3web (version 4.1.0) (https://primer3.ut.ee/) to amplify a genomic region spanning exons 1–3 of the *CtPDS* gene. Polymerase chain reaction (PCR) was performed using gene‐specific primers, and the products were resolved on a 1% agarose gel stained with a safe nucleic acid dye. To determine size, a 1 kb DNA ladder was employed. The resulting PCR amplicon was approximately 1378 bp in length, covering the putative target sites of all three sgRNAs. This amplified region was Sanger sequenced, confirming the accuracy of the *CtPDS* gene annotation and the sgRNA target positions (Figure 2A,B). Subsequently, three sgRNAs (sgRNA1: GTGCTAAGATCACCAAGTTGAGG, sgRNA2: GAGGCAAGAGATGTTCTAGGTGG, and sgRNA3: GATGGAGACTGGTATGAGACGGG) were designed at the exons 1–3, respectively, targeting the *CtPDS* gene using CRISPOR (version 5.2) (Concordet & Haeussler, 2018) and Benchling (version 1.22.0) (www.benchling.com) based on high specificity, cutting efficiency, and low off‐target risk (Table S2).

### 2.6 Plasmid preparation and constructs

The CRISPR/Cas9 expression vector was assembled using three intermediate module plasmids A, B, and C, and a destination plasmid pTRANS_100 (Addgene plasmid #91198), which enables the efficient combination of Cas9 and sgRNA expression cassettes. The Cas9 scaffold was assembled by Golden Gate cloning using the type IIS restriction enzyme (RE) *AarI*, following the protocol described by Čermák et al. (2017). Module A (pMOD_A0101; Addgene #90998), encoding an *Arabidopsis* (*Arabidopsis thaliana*) codon‐optimized SpCas9 (AtCas9) driven by the CaMV35S promoter, was combined with empty Modules B (pMOD_B000) and C (pMOD_C0000; Addgene #91081) into the pTRANS_100 backbone to develop modified pTRANS_100_ AtCas9_no sgRNA. This reaction replaced the ccdB negative selection cassette at the gRNA insertion site with a seamless backbone, yielding an intermediate construct lacking the gRNA cassette.

A polycistronic gRNA cassette encoding three gRNAs targeting the first three exons of the *CtPDS* gene was custom‐synthesized (Genscript Biotech Ltd.) (Figure S2C). The gRNA cassette was PCR‐amplified using primers designed to introduce flanking restriction sites: the forward primer containing the *SphI* site (5′‐AACTGCATGCTGGCAGACATACTGTCCCAC‐3′) and the reverse primer containing the *AscI* site (5′‐CTATGGCGCGCCGATCTGGATTTTAGTACTGGATT‐3′). The PCR product was gel‐purified. Now, both pTRANS_100_ AtCas9_no sgRNA and gRNA cassette fragments were digested with *SphI* and *AscI* REs, gel‐purified, and ligated using T4 DNA ligase (New England Biolabs) at 16°C overnight. This step inserted the gRNA cassette into the vector backbone downstream of the Cas9 scaffold and developed a nonbinary vector (pTRANS_100_*CtPDS*) for protoplast transformation (Figure S3A).

Cloning success was confirmed by diagnostic restriction digestion with *SphI* and *AscI* and agarose gel electrophoresis (Figure S3B). Correct insertion and orientation of the gRNA cassette were verified by Sanger sequencing. The final vector contains a constitutively expressed AtCas9 nuclease and a multiplexed gRNA cassette encoding three gRNAs targeting the first three exons of the *CtPDS* gene, providing a robust platform for targeted genome editing. A schematic representation of the final assembled vector (Figure S3C) was created using SnapGene (version 8.1.1) (www.snapgene.com), providing a visual overview of the construct used for PEG‐mediated protoplast transformation in guar.

### 2.7 Mutation analysis for the protoplast assay

Genomic DNA was extracted from transformed guar protoplasts after 24 h post‐transformation under dark conditions using the cetyltrimethylammonium bromide method (Doyle & Doyle, 1987). The same primer pair used for *CtPDS* gene validation (spanning exons 1–3) was employed to amplify the CRISPR target region for mutation analysis. PCR was carried out using the KAPA3G PCR kit (Kapa Biosystems) under the following thermal cycling conditions: initial denaturation at 95°C for 3 min; 40 cycles of denaturation at 95°C for 30 s, annealing at 58°C for 30 s, and extension at 72°C for 45 s; followed by a final extension at 72°C for 3 min. Each PCR reaction included a negative control (NC) to confirm the absence of contamination. PCR products were purified by gel extraction and cloned into a TOPO vector (Thermo Fisher Scientific). Mutations at the *CtPDS* target sites were identified by Sanger sequencing.

### 2.8 Statistical analysis

The experiment was carried out using a completely randomized design with three independent replicates (*n* = 3) per treatment. To analyze the data, we used one‐way analysis of variance (ANOVA) in Statistix 10 (version 10.0.1.5) (www.statistix.com), and treatment means were compared using the least significant difference (LSD) test at a significance threshold of *p* < 0.05. Graphs were generated in R (RStudio version 2025.05.1+513) using the “ggplot2” and “ggpubr” packages. Bar plots show treatment means ± standard deviation with individual replicate values displayed as jittered points, and treatment lettering was assigned based on LSD post hoc comparisons.

## 3 RESULTS

### 3.1 Effect of seedling age and tissue type on protoplast isolation in guar

**FIGURE 1:**
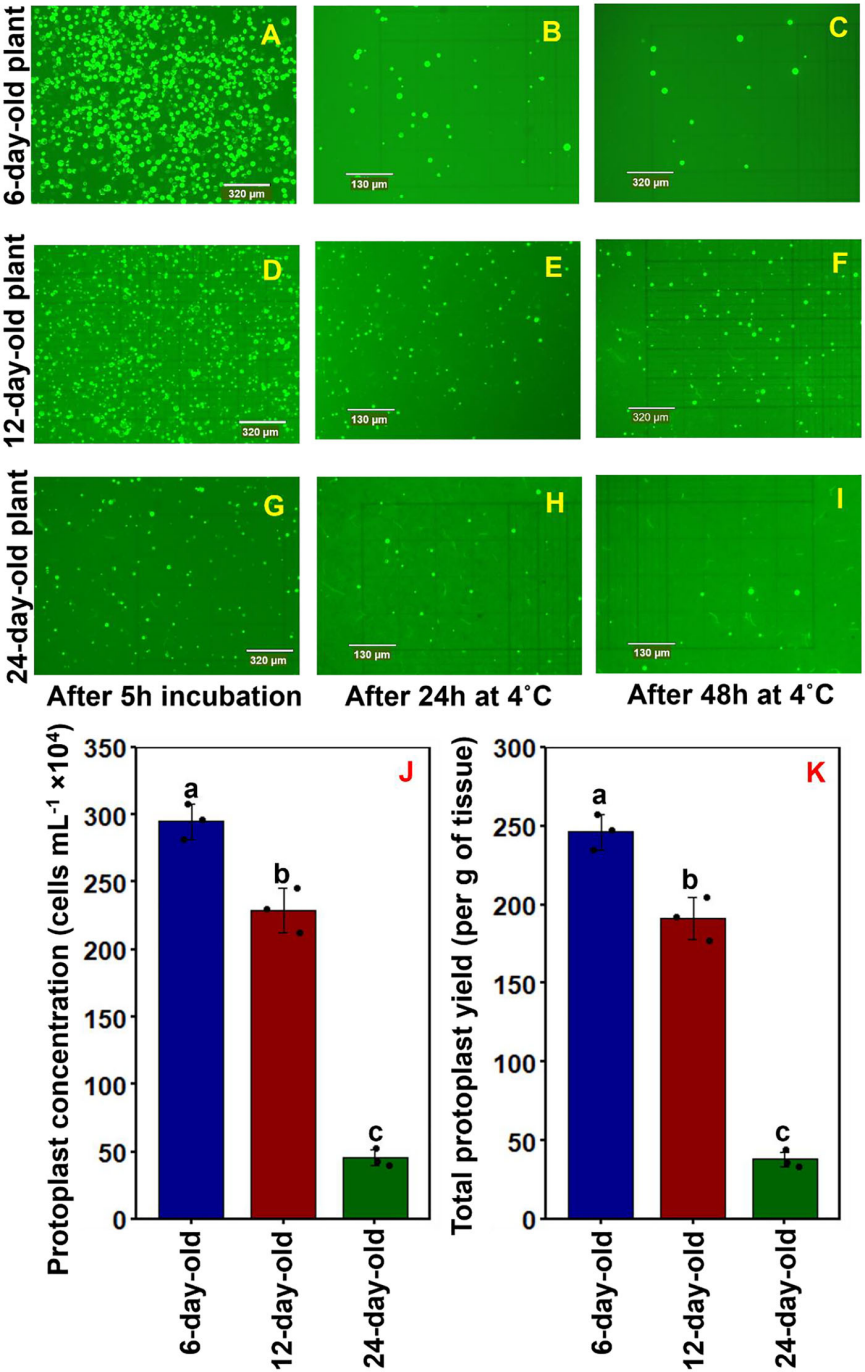
Protoplast isolation from different tissues and ages of guar seedlings. (A–C) Fluorescent micrographs of protoplasts from 6‐day‐old cotyledons after 5 h incubation, 24 h at 4°C, and 48 h at 4°C, respectively. (D–F) Protoplasts from 12‐day‐old unifoliate leaves after 5 h incubation, 24 h at 4°C, and 48 h at 4°C, respectively. (G–I) Protoplasts from 24‐day‐old trifoliate leaves after 5 h incubation, 24 h at 4°C, and 48 h at 4°C, respectively. (J) Bar graph showing protoplast concentration (cells mL^−1^ × 10⁴). (K) Bar graph showing total protoplast yield (per g of tissue). Data represent mean ± standard deviation from three biological replicates (*n* = 3). Different letters indicate statistically significant differences at *p* < 0.05, according to least significant difference (LSD) test.

To determine the optimal tissue source for protoplast isolation in guar, seedlings at three developmental stages, 6‐day‐old cotyledons, 12‐day‐old unifoliate leaves, and 24‐day‐old trifoliate leaves, were evaluated (Figure 1A–I). Protoplasts were examined immediately after 5 h of incubation, 24 h at 4°C, and 48 h at 4°C. Microscopic observations showed that 6‐day‐old cotyledons produced abundant, spherical, and largely intact protoplasts (Figure 1A–C), while 12‐day‐old unifoliate leaves yielded moderately abundant, intact protoplasts (Figure 1D–F), and 24‐day‐old trifoliate leaves yielded few protoplasts with noticeable cellular debris (Figure 1G–I).

Quantitative analysis confirmed that both protoplast concentration (cells mL^−1^ × 10⁴) and total yield (per g of tissue) were significantly higher in 6‐day‐old cotyledons (∼295 and ∼246), compared with older tissues (Figure 1J,K). Relative to 6‐day‐old seedlings, total yield from 12‐day‐old leaves decreased by 22.26%, whereas 24‐day‐old leaves showed a substantial reduction of 84.55%. These results indicate that 6‐day‐old cotyledons represent the most suitable tissue source for guar protoplast isolation, providing high yield and cellular integrity for downstream transformation experiments.

### 3.2 Effect of different isolation methods on protoplast isolation in guar

**FIGURE 2:**
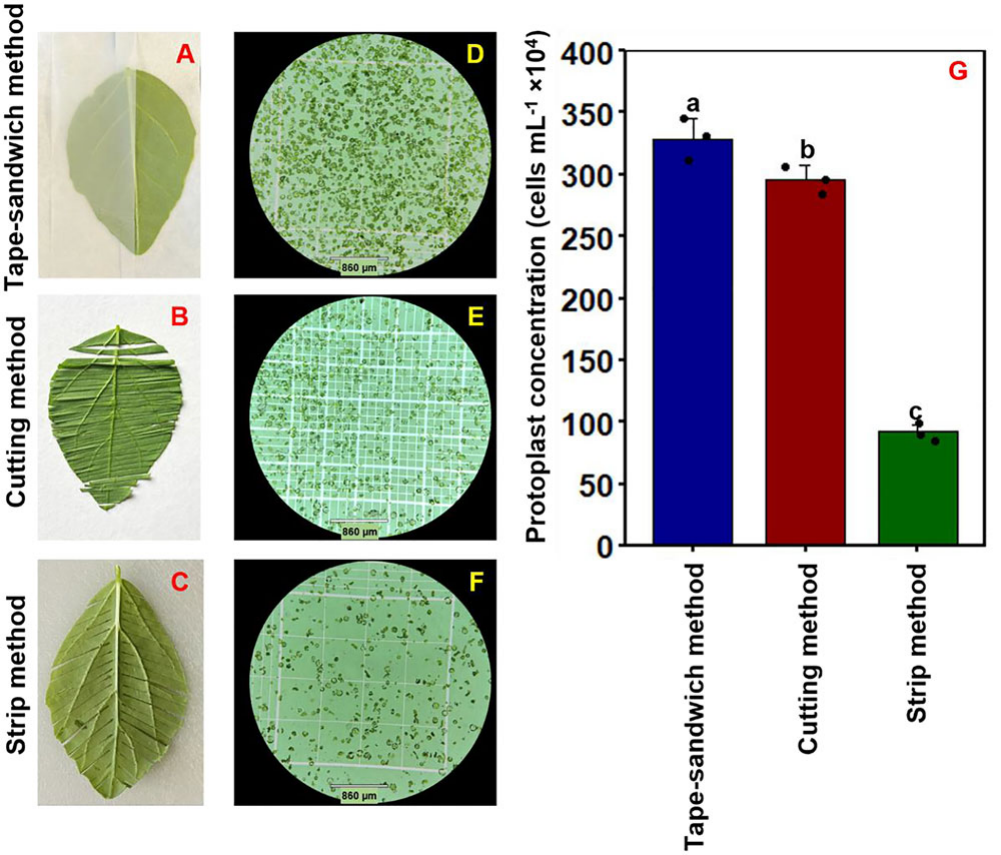
Optimization of protoplast isolation methods in guar seedlings. (A–C) Representative images of the tape‐sandwich, cutting, and strip methods, respectively. (D–F) Bright‐field micrographs showing protoplasts obtained using each method. (G) Bar graph showing protoplast concentration (cells mL^−1^ × 10⁴) for the three methods. Data represent mean ± standard deviation from three biological replicates (*n* = 3). Different letters indicate statistically significant differences at *p* < 0.05, according to least significant difference (LSD) test.

To optimize protoplast isolation in guar, we compared three methods: tape‐sandwich, cutting, and strip (Figure 2A–C). BF imaging revealed that the tape‐sandwich method yielded the highest number of intact and spherical protoplasts. In contrast, the cutting method produced fewer protoplasts with some cellular debris, and the strip method yielded the lowest number of protoplasts (Figure 2D–F). Quantitative analysis confirmed that protoplast concentration (cells mL^−1^ × 10⁴) was significantly higher using the tape‐sandwich method (∼329), followed by the cutting method (∼296), which was considerably higher than the strip method (∼92) (Figure 2G). Relative to the tape‐sandwich method, protoplast concentration decreased by 10.03% in the cutting method and by 72.04% in the strip method, indicating that the tape‐sandwich method is the most effective and reliable approach for guar protoplast isolation.

### 3.3 Effect of cellulase concentration methods on protoplast isolation in guar

**FIGURE 3:**
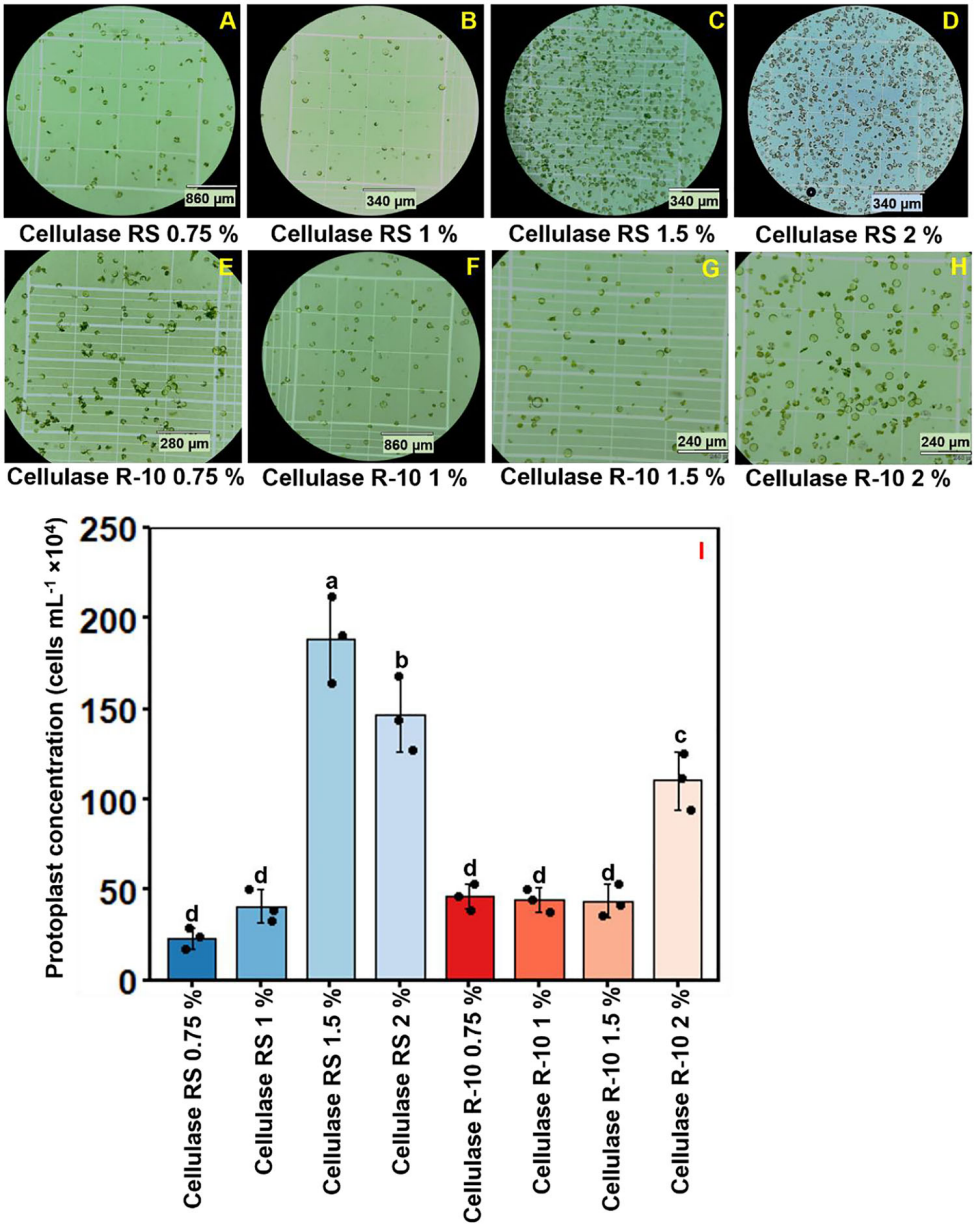
Optimization of cellulase concentration for protoplast isolation in guar seedlings. (A–D) Bright‐field images of protoplasts obtained with cellulase RS at 0.75%, 1%, 1.5%, and 2%, respectively. (E–H) Bright‐field images of protoplasts obtained with cellulase R‐10 at 0.75%, 1%, 1.5%, and 2%, respectively. (I) Bar graph showing protoplast concentration (cells mL^−1^ × 10⁴) for all tested concentrations. Data represent mean ± standard deviation from three biological replicates (*n* = 3). Different letters indicate statistically significant differences at *p* < 0.05, according to least significant difference (LSD) test.

To optimize enzyme conditions for protoplast isolation in guar, we tested two cellulase types, RS and R‐10, at four concentrations each (0.75%, 1%, 1.5%, and 2%) (Figure 3A–H). BF images showed differences in protoplast abundance and integrity across treatments. Protoplast concentration (cells mL^−1^ × 10⁴) was significantly affected by enzyme type and concentration (Figure 3I). The highest yield was obtained with 1.5% cellulase RS (∼189), followed by 2% cellulase RS (∼146) and 2% cellulase R‐10 (∼110). Other concentrations did not differ significantly. Compared with 1.5% cellulase RS, protoplast concentration decreased by 87.63% with 0.75% RS, 78.27% with 1% RS, 22.26% with 2% RS, 75.44% with 0.75% R‐10, 76.33% with 1% R‐10, 76.68% with 1.5% R‐10, and 41.34% with 2% R‐10, indicating that 1.5% cellulase RS is the optimal enzyme condition for guar protoplast isolation.

### 3.4 Effect of mannitol concentration and pectinase on protoplast isolation in guar

**FIGURE 4:**
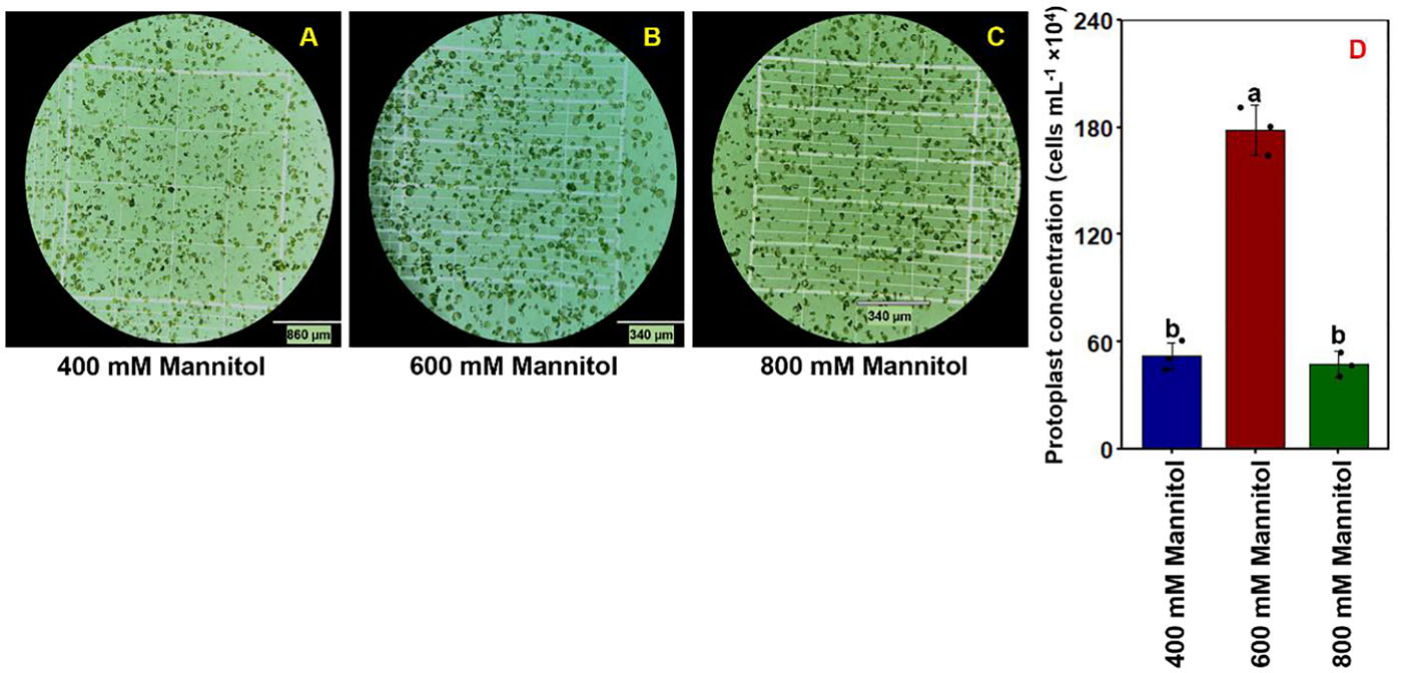
Optimization of mannitol concentration for protoplast isolation in guar seedlings. (A–C) Bright‐field images of protoplasts isolated using 400, 600, and 800 mM mannitol, respectively. (D) Bar graph showing protoplast concentration (cells mL^−1^ × 10⁴) for all tested concentrations. Data represent mean ± standard deviation from three biological replicates (*n* = 3). Different letters indicate statistically significant differences at *p* < 0.05, according to least significant difference (LSD) test.

**FIGURE 5:**
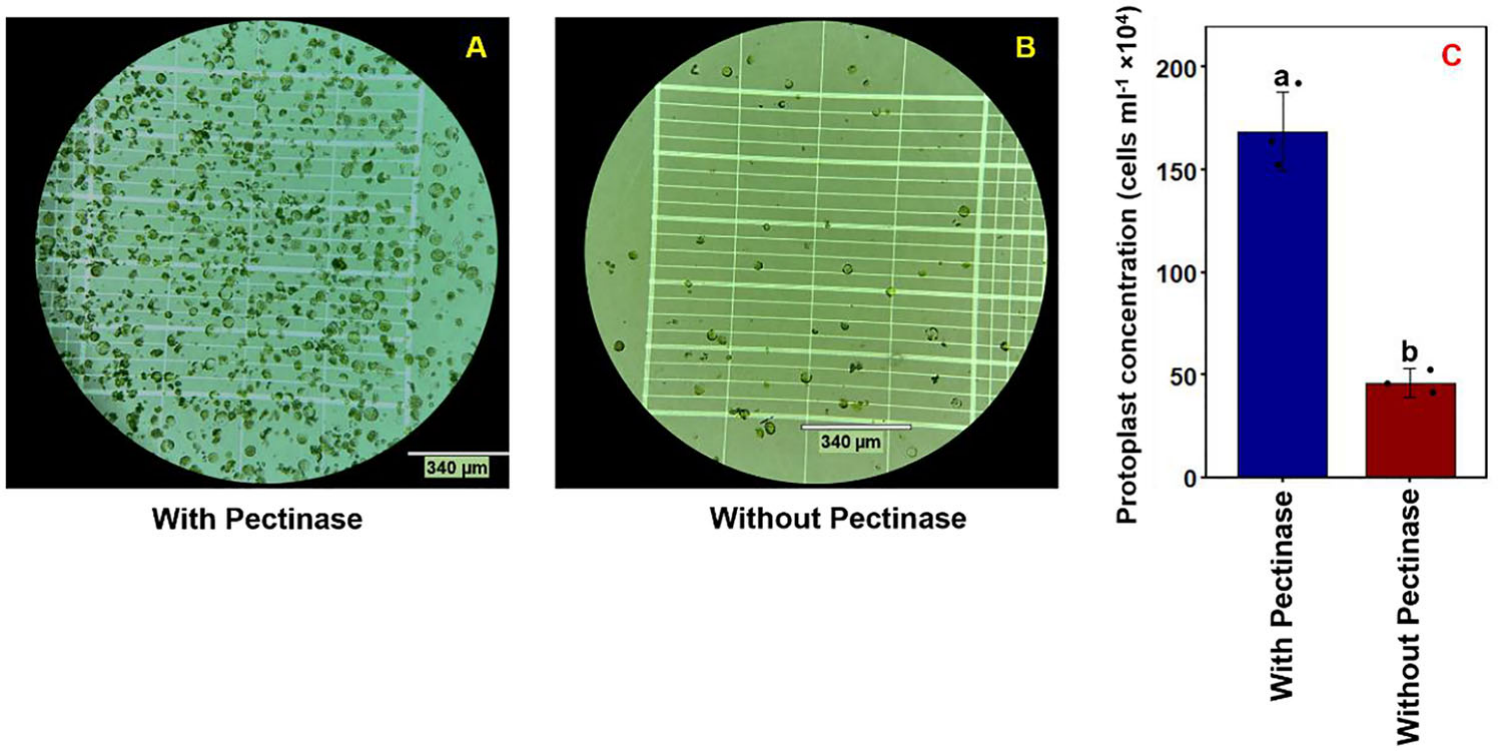
Effect of pectinase on protoplast isolation in guar seedlings. (A, B) Bright‐field images of protoplasts isolated with and without pectinase. (C) Bar graph showing protoplast concentration (cells mL^−1^ × 10⁴) for comparing the presence and absence of pectinase. Data represent mean ± standard deviation from three biological replicates (*n* = 3). Different letters indicate statistically significant differences at *p* < 0.05, according to least significant difference (LSD) test.

To optimize osmotic conditions for guar protoplast isolation, we tested three mannitol concentrations (400, 600, and 800 mM) and evaluated the effect of pectinase in the isolation buffer (Figures 4 and 5). BF microscopy revealed that 600 mM mannitol maintained intact protoplast integrity, whereas 400 and 800 mM caused osmotic imbalance, leading to cell bursting and increased debris. Protoplast concentration at 600 mM mannitol was significantly higher (∼179), while 400 and 800 mM showed reductions of approximately 70.9% and 73.5%, respectively, compared to 600 mM. Similarly, inclusion of 0.3% (w/v) pectinase significantly improved protoplast yield, with a 72.7% reduction observed in its absence. These results indicate that 600 mM mannitol combined with pectinase represents the optimal condition for high‐yield, intact protoplast isolation in guar.

### 3.5 Effect of vacuum infiltration on protoplast isolation in guar

**FIGURE 6:**
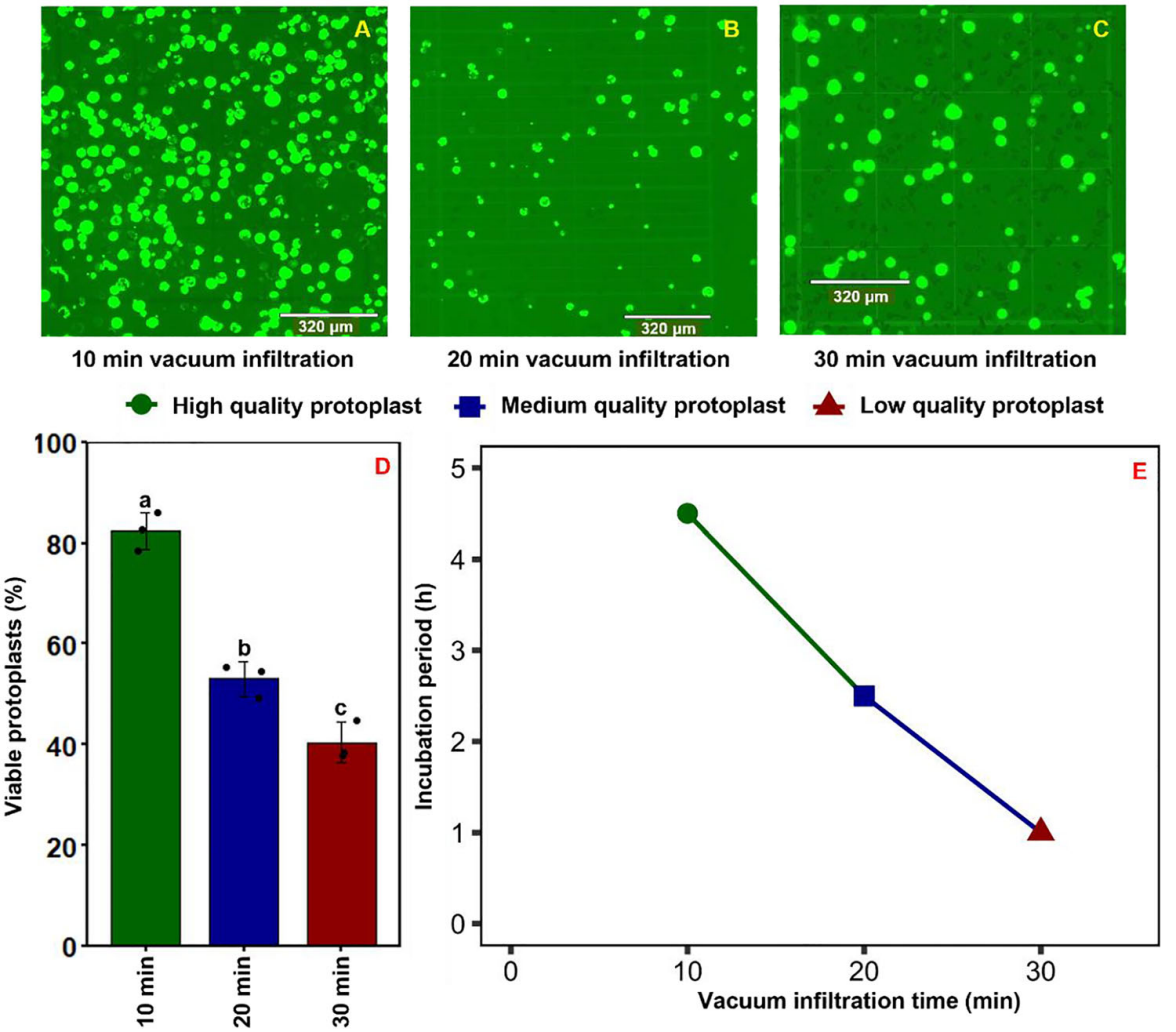
Effect of vacuum infiltration on protoplast viability in guar seedlings. (A–C) Fluorescent microscopy images of protoplasts isolated after 10, 20, and 30 min of vacuum infiltration, respectively. (D) Bar graph showing the percentage of viable protoplasts, highlighting a significant reduction with increasing vacuum duration. (E) Line chart illustrating the relationship between vacuum infiltration time (min), incubation period (h), and protoplast quality (high, medium, and low). Data represent mean ± standard deviation from three biological replicates (*n* = 3). Different letters indicate statistically significant differences at *p* < 0.05, according to least significant difference (LSD) test.

To optimize protoplast viability, we tested three vacuum infiltration durations (10, 20, and 30 min) prior to isolation (Figure 6). FL microscopy revealed that 10 min of vacuum infiltration produced the highest proportion of viable protoplasts, whereas 20 and 30 min of treatment reduced viability significantly. Quantitative analysis showed that 20 and 30 min of vacuum infiltration reduced viable protoplast percentage by approximately 35.5% and 50.8%, respectively, compared to 10 min. Line chart analysis indicated that longer vacuum infiltration correlated with shorter incubation times to achieve optimal protoplast quality. 10 min of vacuum infiltration required ∼4.5 h, yielding high‐quality intact protoplasts, while 20 min of infiltration required ∼2.5 h, yielding medium‐quality protoplasts; and 30 min of infiltration required ∼1 h, yielding low‐quality protoplasts. These results indicate that 10 min of vacuum infiltration is optimal for obtaining high‐quality and viable guar protoplasts.

### 3.6 Effect of promoter type and temperature on protoplast transformation in guar

**FIGURE 7:**
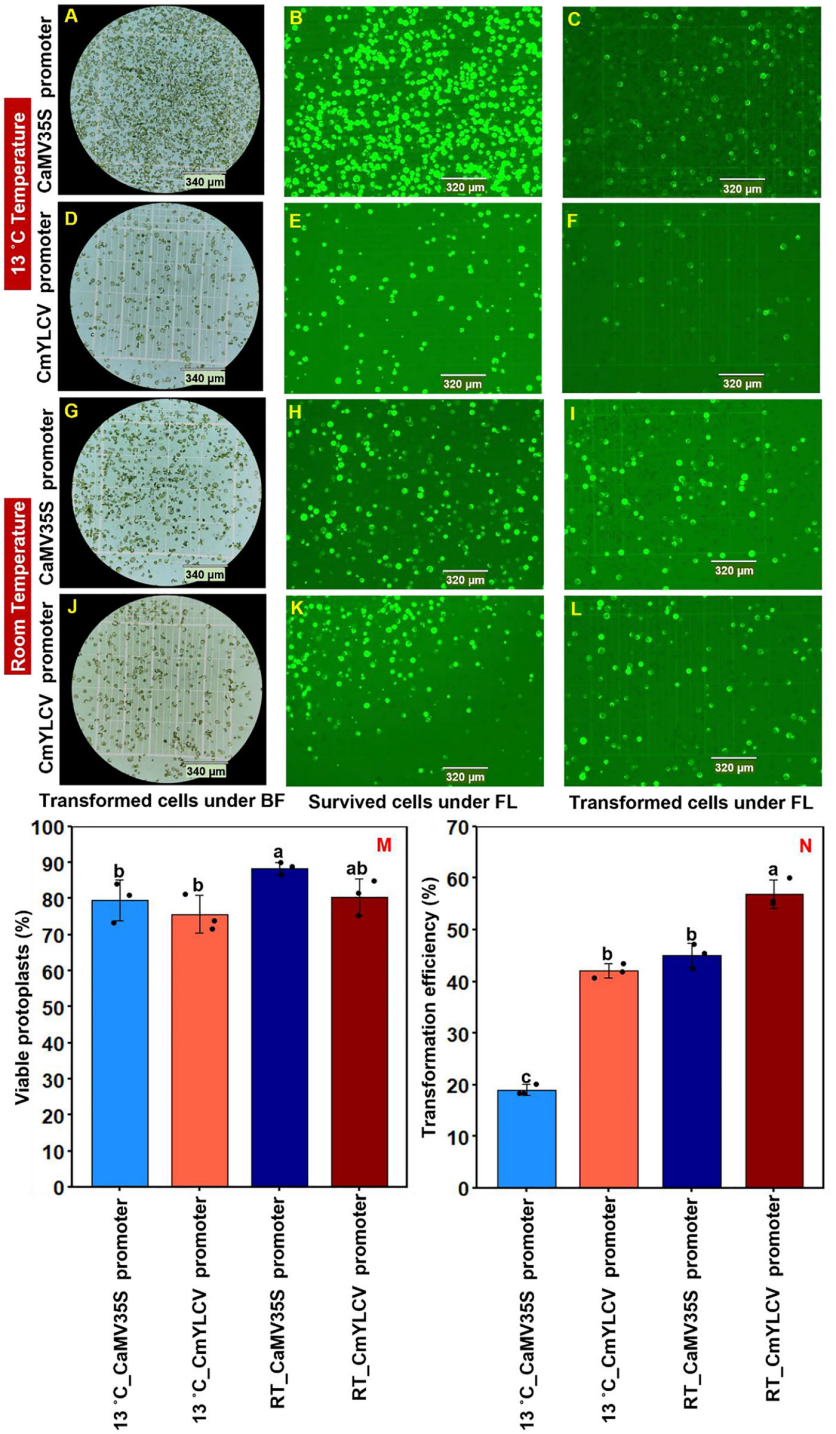
Effect of promoter type and temperature on guar protoplast transformation. (A–C) Bright‐field and fluorescent images of protoplasts transformed with cauliflower mosaic virus 35S promoter (CaMV35S) at 13°C. (D–F) Bright‐field and fluorescent images of protoplasts transformed with cestrum yellow leaf curling virus promoter (CmYLCV) at 13°C. (G–I) Bright‐field and fluorescent images of protoplasts transformed with CaMV35S at RT. (J–L) Bright‐field and fluorescent images of protoplasts transformed with CmYLCV at room temperature (RT). (M) Bar graph showing protoplast viability (%) under different promoter and temperature combinations. (N) Bar graph showing transformation efficiency (%) across the same conditions. Data represent mean ± standard deviation from three biological replicates (*n* = 3). Different letters indicate statistically significant differences at *p* < 0.05, according to least significant difference (LSD) test. BF, bright‐field; FL, fluorescent.

We tested two constitutive promoters (CaMV35S and CmYLCV) for the GFP expression at two temperatures (13°C and RT) to evaluate their effect on guar protoplast transformation (Figure 7). FL microscopy confirmed a greater number of transformed cells with CmYLCV at RT (Figure 7L) compared to CaMV35S at the same temperature (Figure 7I) or either promoter at 13°C (Figure 7C,F). At RT, CmYLCV achieved the highest transformation efficiency, while CaMV35S showed the highest protoplast viability. However, the difference in viability between CaMV35S and CmYLCV at RT was not statistically significant, whereas both RT treatments performed significantly better than their respective 13°C counterparts. Quantitative analysis confirmed that transformation efficiency with CmYLCV at RT was superior (∼56.9%), being 66.6% higher than CaMV35S at 13°C, 26.1% higher than CmYLCV at 13°C, and 20.7% higher than CaMV35S at RT. For viability, CaMV35S at RT was the highest, but its difference from CmYLCV at RT was not significant, whereas reductions of 10.1% and 14.4% were observed for CaMV35S and CmYLCV at 13°C, respectively. Taken together, these results indicate that CmYLCV at RT provides the most effective balance of high transformation efficiency and protoplast viability in guar.

### 3.7 Effect of PEG concentration and incubation time on protoplast transformation in guar

**FIGURE 8:**
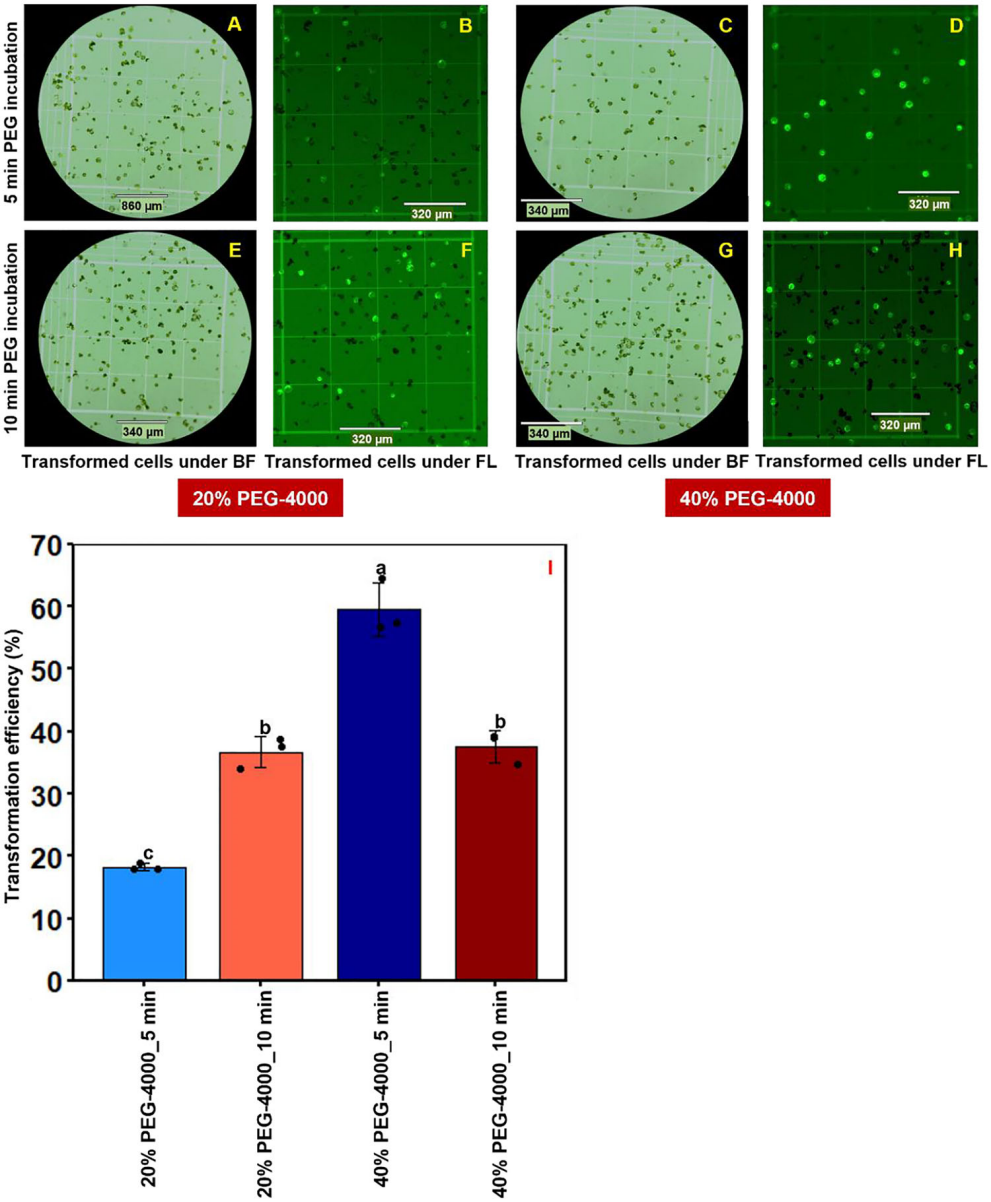
Effect of polyethylene glycol (PEG) concentration and incubation time on guar protoplast transformation. (A and B) Bright‐field and fluorescent images of protoplasts transformed with 20% PEG‐4000 for 5 min. (C and D) Bright‐field and fluorescent images of protoplasts transformed with 40% PEG‐4000 for 5 min. (E and F) Bright‐field and fluorescent images of protoplasts transformed with 20% PEG‐4000 for 10 min. (G and H) Bright‐field and fluorescent images of protoplasts transformed with 40% PEG‐4000 for 10 min. (I) Bar graph showing transformation efficiency (%) for all four treatments. Data represent mean ± standard deviation from three biological replicates (*n* = 3). Different letters indicate statistically significant differences at *p* < 0.05, according to least significant difference (LSD) test. BF, bright‐field; FL, fluorescent.

To optimize transformation parameters, we tested two concentrations of PEG‐4000 (20% and 40%) and two incubation times (5 and 10 min) (Figure 8). BF and FL microscopy confirmed transformed protoplasts under all conditions. Quantitative analysis revealed that 40% PEG with 5 min incubation produced the highest transformation efficiency (∼59.5%). Compared to this optimal condition, transformation efficiency decreased by 69.3% with 20% PEG for 5 min, 38.3% with 20% PEG for 10 min, and 36.8% with 40% PEG for 10 min. These results demonstrate that a short incubation with higher PEG concentration (40% PEG, 5 min) provides the most effective condition for guar protoplast transformation.

### 3.8 Validation of *CtPDS* gene amplification by PCR and sequencing

To confirm successful amplification of the target *CtPDS* gene, PCR was performed using gene‐specific primers. A single clear band of the expected size (701 bp in Sample 1 and 1446 bp in Sample 2) was observed (Figure S4). NC1 and NC2 showed no amplifications, confirming the absence of contamination. The lack of nonspecific bands or smearing indicated high specificity of primer binding and good template DNA quality, which was further confirmed by sequencing of the *CtPDS* gene from wild type (WT) genomic DNA, showing 100% identity with the reference sequence. These results confirmed that the designed primers reliably amplified the target *CtPDS* fragments, suitable for downstream CRISPR/Cas9 validation assays.

### 3.9 Mutation analysis of *CtPDS* gene editing in guar protoplasts

**FIGURE 9:**
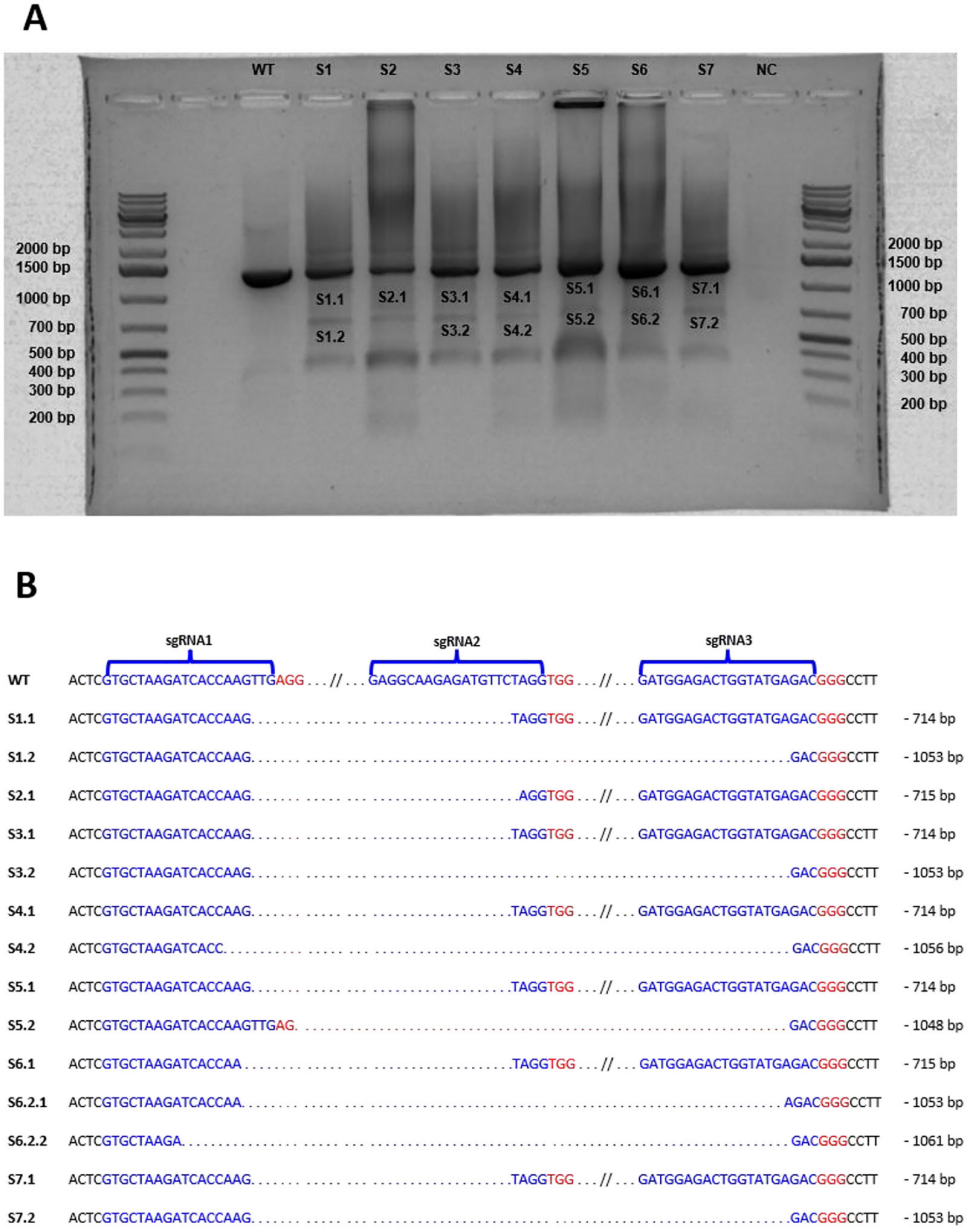
Clustered regularly interspaced short palindromic repeats/CRISPR‐associated protein 9 (CRISPR/Cas9)‐mediated *Cyamopsis tetragonoloba phytoene desaturase* (*CtPDS*) gene editing in guar protoplasts. (A) Agarose gel showing polymerase chain reaction (PCR) amplification of the *CtPDS* locus. (B) Summary of observed deletions at the *CtPDS* target site for each transformed sample, highlighting fragment sizes and editing patterns. NC, negative control; S1–S7, transformed protoplast samples; sgRNA, single guide RNA; WT, wild‐type control; 1 kb DNA ladder as size marker.

To assess the gene‐editing efficiency of the CRISPR/Cas9 vector targeting *CtPDS*, guar protoplasts were transformed using the optimized PEG‐mediated protocol. Genomic DNA was extracted from seven transformed samples, and PCR amplification of the target locus was performed (Figure 9). Three distinct bands were observed in the edited samples. The upper band corresponded to the WT unedited fragment, while the middle and lower bands represented edited fragments resulting from CRISPR/Cas9‐induced deletions. The middle edited fragment (∼714–715 bp) resulted from simultaneous cleavage by sgRNA1 and sgRNA2, whereas the lower edited fragment (∼1048–1061 bp) indicated larger deletions involving all sgRNA target sites. No amplification was detected in NCs, and the WT produced the expected full‐length fragment. All seven transformed samples showed edited bands, corresponding to a 100% editing efficiency. These results confirm the high efficiency and reliability of the optimized protoplast‐based genome editing system in guar.

## 4 DISCUSSION

Protoplast isolation is a fundamental technique that enables genetic manipulation and functional studies by providing plant cells devoid of cell walls, facilitating direct DNA uptake and genome editing (Yue et al., 2021). In this study, we established a robust protocol for protoplast isolation from guar through systematic evaluation of seedling age, tissue type, isolation method, and enzymatic conditions. Our results demonstrate that 6‐day‐old cotyledons yield the highest number of intact and viable protoplasts. In contrast, older unifoliate and trifoliate leaves show progressively lower yields and increased cellular debris, highlighting the critical influence of developmental stage on protoplast quality (Figure 1). This trend reflects age‐dependent changes in cell wall composition and tissue rigidity. Younger cotyledons have thinner, less lignified walls and greater cellular plasticity, facilitating enzymatic digestion and efficient protoplast release. In contrast, older unifoliate and trifoliate leaves develop more rigid walls, which limit enzyme access and reduce yield and integrity (Jahan et al., 2023; Song et al., 2021). These observations align with previous findings in legumes such as chickpea, cowpea, and peanut, where juvenile tissues consistently yield higher protoplasts and exhibit higher viability (Biswas, Bridgeland, et al., 2022; Biswas, Wahl, et al., 2022; Bridgeland et al., 2023; Irum et al., 2025), highlighting the importance of selecting young tissues for downstream applications.

Among the isolation methods evaluated, the tape‐sandwich technique yielded superior protoplast concentration and integrity in guar (Figure 2). Its effectiveness is attributable to maximal mesophyll exposure with minimal mechanical stress, which promotes uniform enzyme penetration while preserving plasma membrane integrity (Wu et al., 2009; Yue et al., 2021). In contrast, the cutting and strip methods introduce localized wounding and heterogeneous enzyme access, resulting in increased cellular debris and reduced yield. This mechanistic rationale aligns with prior observations in legumes and *Arabidopsis*, where gentle tissue handling and enhanced surface contact significantly improve protoplast recovery and viability (Bridgeland et al., 2023; Reed & Bargmann, 2021; Yoo et al., 2007).

Enzymatic digestion conditions were critically optimized, revealing that 1.5% cellulase RS combined with pectinase in a 600 mM mannitol buffer provided maximal protoplast yield and structural integrity (Figure 3, 4, 5). This balance ensures efficient depolymerization of cellulose and hemicellulose, as well as the targeted degradation of pectin in the middle lamella of cell walls, facilitating protoplast release without inducing osmotic stress or membrane damage (Kim et al., 2025; Wang et al., 2022; Wiszniewska & Pindel, 2009). Lower enzyme concentrations were insufficient to degrade the rigid cell wall, while higher concentrations or inappropriate osmotic conditions caused plasmolysis or cell collapse, reducing viability (Potrykus & Shillito, 1986). The inclusion of pectinase together with an optimal concentration of mannitol maintained osmotic balance, preventing protoplast bursting or shrinkage. This principle has been demonstrated in protoplast isolations from *Vitis* species as well as legumes such as cowpea and peanut (Bridgeland et al., 2023; Tricoli & Debernardi, 2024; Biswas, Bridgeland, et al., 2022). These results highlight the intricate interplay between enzymatic activity and osmotic stabilization in maintaining protoplast integrity during the isolation process.

Vacuum infiltration for 10 min prior to protoplast isolation significantly enhanced yield and viability (Figure 6) by removing intercellular air pockets, thereby promoting uniform enzyme penetration into the tissue matrix. This short‐term infiltration facilitates rapid digestion while preserving plasma membrane integrity (Cheng & Nakata, 2020; MacDonald, 1975; Nanjareddy et al., 2016; Shan et al., 2014; Xue et al., 2023). Conversely, extended vacuum exposure had a detrimental effect on protoplast quality, presumably through mechanical stress and excessive turgor loss, which destabilized the plasma membrane and induced cell lysis, consistent with the findings by Li et al. (2022). These findings highlight the critical balance between improving enzyme access and maintaining cellular integrity, corroborating previous observations in legumes and model species where optimized vacuum conditions are essential for high‐quality protoplast recovery.

Both the promoter type and incubation temperature strongly influenced protoplast transformation efficiency. The CmYLCV promoter at RT yielded the highest transient expression in guar (Figure 7), reflecting its inherently strong transcriptional activity and broad expression profile in dicot systems, likely due to cis‐regulatory elements favoring robust transcription (Sahoo et al., 2014; Stavolone et al., 2003). RT conditions further support optimal cellular metabolism, enhancing promoter activity without significantly compromising viability. In contrast, CaMV35S maintained slightly higher, though not statistically significant, protoplast viability, indicating that transformation efficiency is primarily driven by promoter strength rather than cytotoxic effects. These results align with previous studies in other legumes, including chickpea and peanut, highlighting the suitability of CmYLCV for high‐efficiency transient expression in protoplast‐based functional assays (Biswas, Wahl, et al., 2022; Irum et al., 2025).

PEG‐mediated transformation was optimized using 40% PEG for a 5‐min incubation, which efficiently induced transient membrane permeability for DNA uptake while minimizing cytotoxic effects associated with prolonged exposure (Figure 8). This brief, high‐concentration treatment ensures effective genetic delivery without compromising protoplast integrity, consistent with established PEG‐based transformation protocols in diverse plant species (Bridgeland et al., 2023; Shao et al., 2023; Yang et al., 2022).

Molecular validation through PCR amplification of the *CtPDS* target gene confirmed both the specificity of the designed primers and the integrity of genomic DNA extracted from transformed guar protoplasts, ensuring suitability for downstream CRISPR/Cas9 analyses (Figure 9). Subsequent mutation detection revealed distinct deletions within the *CtPDS* locus, characteristic of non‐homologous end joining repair following Cas9‐mediated cleavage (Biswas, Wahl, et al., 2022; Bortesi & Fischer, 2015). The presence of multiple edited fragments, contrasting with wild‐type bands, provides direct evidence of successful targeted gene disruption. These mutational patterns and editing efficiencies are consistent with CRISPR‐mediated *PDS* editing reported in legumes and other crop species (Bridgeland et al., 2023; Mainkar et al., 2023; Odipio et al., 2017; Prasad et al., 2024), establishing a reliable protoplast‐based platform for transient functional genomics and genome editing in guar. However, stable transformation remains challenging due to low regeneration efficiency and genotype dependence, as reported in previous *Agrobacterium*‐mediated studies (Joersbo, 2003; Joersbo et al., 1999, 2001). We believe that our optimized protoplast platform provides a strong foundation for future development of gene‐editing protocols in guar, as it enables rapid validation of sgRNAs before moving into the resource‐ and time‐intensive stable transformation.

## 5 CONCLUSION

In this study, we established the first optimized protoplast‐based genome editing system for guar, utilizing 6‐day‐old cotyledons and the tape‐sandwich method to ensure high protoplast yield and viability. Optimized enzyme digestion, osmotic balance, and PEG‐mediated transformation, facilitated by the CmYLCV promoter, enabled efficient transient expression. CRISPR/Cas9 editing of *CtPDS* further validated this platform, achieving 100% editing efficiency in transformed protoplast samples, providing a reliable framework to accelerate functional genomics and targeted breeding in guar.

## AUTHOR CONTRIBUTIONS

**Protik Kumar Ghosh**: Data curation; formal analysis; methodology; software; writing—original draft; writing—review and editing. **Sudip Biswas**: Data curation; investigation; methodology; validation; writing—review and editing. **Roly Malaker**: Investigation; methodology. **Hanh Pham**: Writing—review and editing. **Endang M. Septiningsih**: Conceptualization; methodology; project administration; resources; supervision; validation; writing—review and editing. **Waltram Ravelombola**: Conceptualization; funding acquisition; resources; supervision; writing—review and editing.

## CONFLICT OF INTEREST STATEMENT

The authors declare no conflicts of interest.

## Supporting information



Supplementary Figure 1. Phylogenetic analysis of *PDS* genes in selected legume species.
Supplementary Figure 2. Design of sgRNAs targeting *CtPDS* gene.
Supplementary Figure 3. CRISPR/Cas9 vector constructs developed for *CtPDS* gene editing in guar.
Supplementary Figure 4. Validation of *Cyamopsis tetragonoloba phytoene desaturase* gene amplification in guar by PCR.
Supplementary Table 1. BLAST alignment results of 13 soybean *PDS* exons against the guar genome.
Supplementary Table 2. sgRNAs targeting *CtPDS* exons with their sequences, specificity scores, on‐target efficiency, out‐of‐frame prediction, Lindel score, and off‐target information.

## Data Availability

All data are available within the published paper and Supporting Information.
