# Supplementary material for: Optimization of CRISPR/Cas9‐mediated CtPDS knockout in guar protoplasts
Source: Plant Genome. 2026 Feb 11;19(1):e70177. doi: 10.1002/tpg2.70177 (PMC12895976; doi:10.1002/tpg2.70177)
Supplement: Supplementary file 1 [file TPG2-19-e70177-s001.pdf]

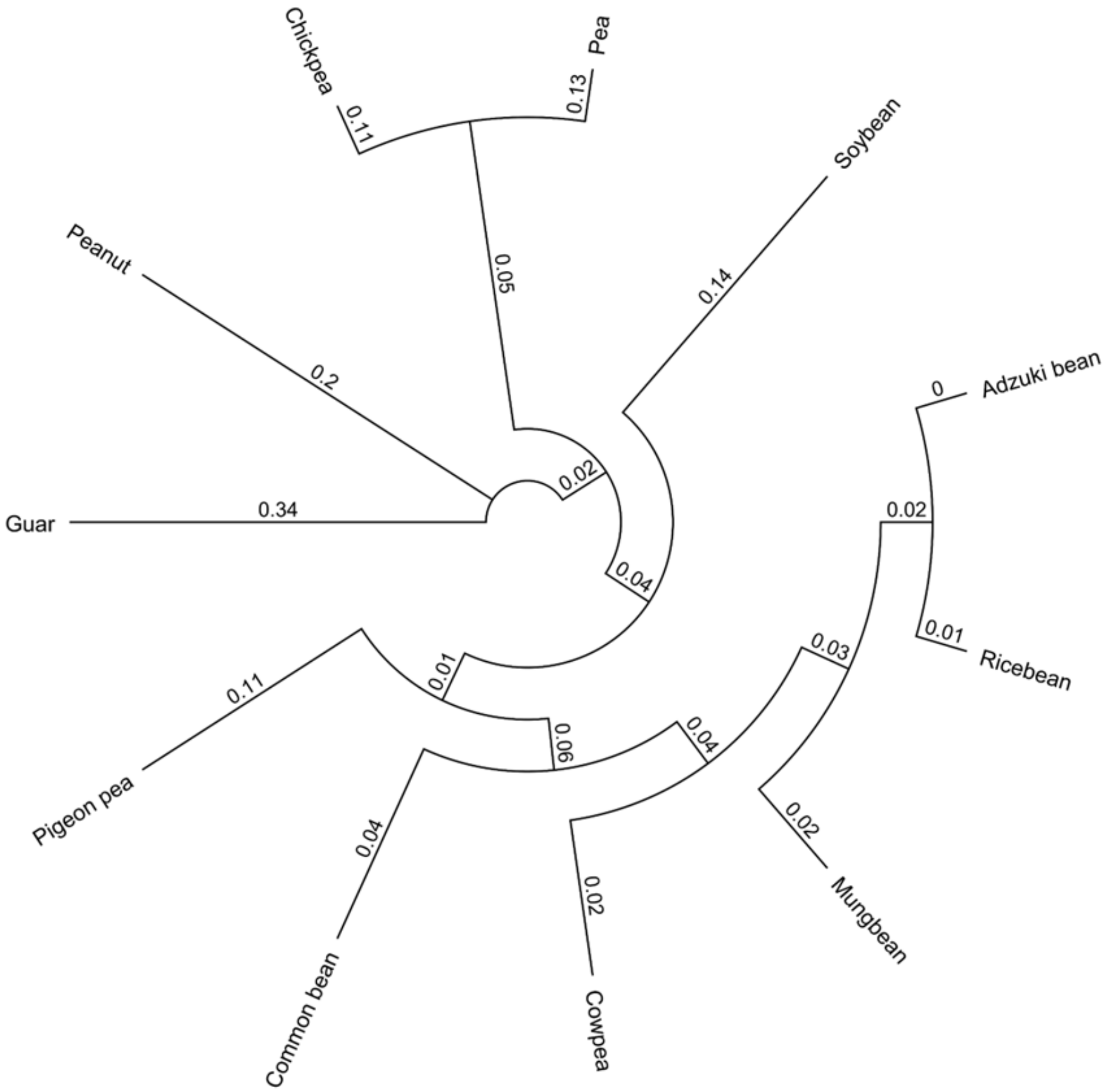

SUPPLEMENTARY FIGURE 1. Phylogenetic analysis of PDS genes in selected legume species. The phylogenetic tree was constructed using PDS gene sequences from chickpea, pea, soybean, adzuki bean, rice bean, mungbean, cowpea, common bean, pigeonpea, peanut, and guar. Branch-level values indicate the support for each node, illustrating the evolutionary relationships among these legume species.

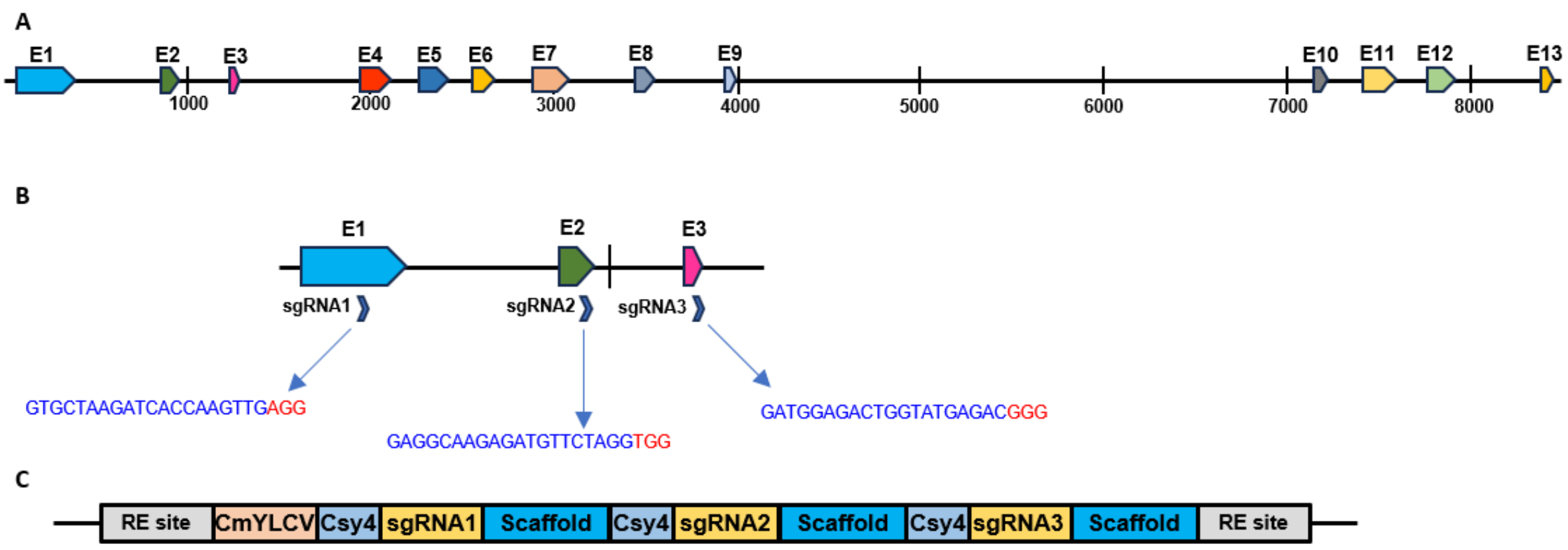

SUPPLEMENTARY FIGURE 2. Design of sgRNAs targeting *CtPDS* gene. (A) Schematic representation of the *CtPDS* gene showing all 13 exons. (B) Zoomed view of the first three exons, highlighting the locations of the designed sgRNAs with arrows and their corresponding target sequences. (C) Schematic of the CmYLCV-Csy4-gRNA cassette. *CtPDS*, *Cyamopsis tetragonoloba phytoene desaturase*; E1-E13, exon 1 to exon 13 in *CtPDS* gene; sgRNA, single-guide RNA.

A

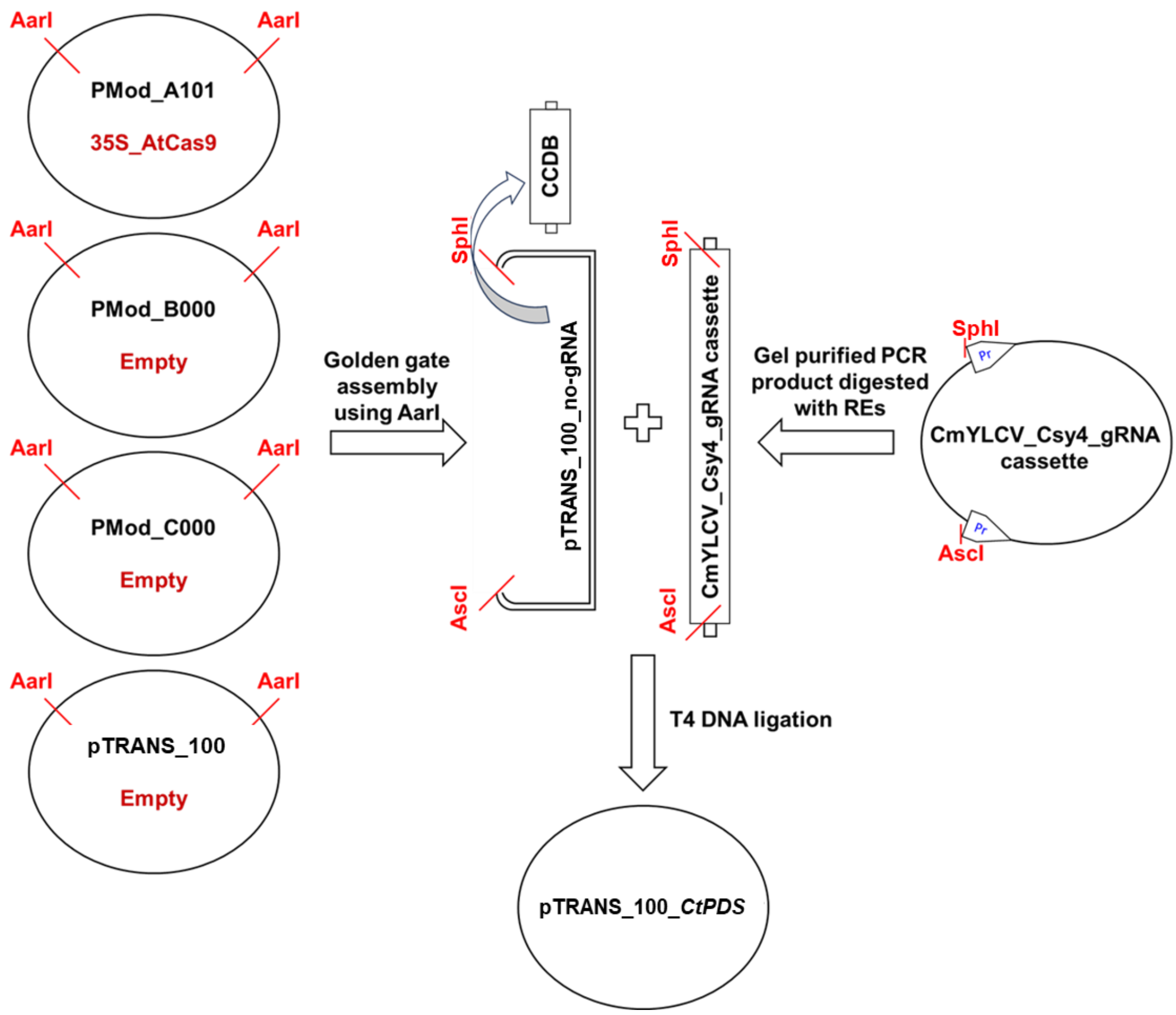

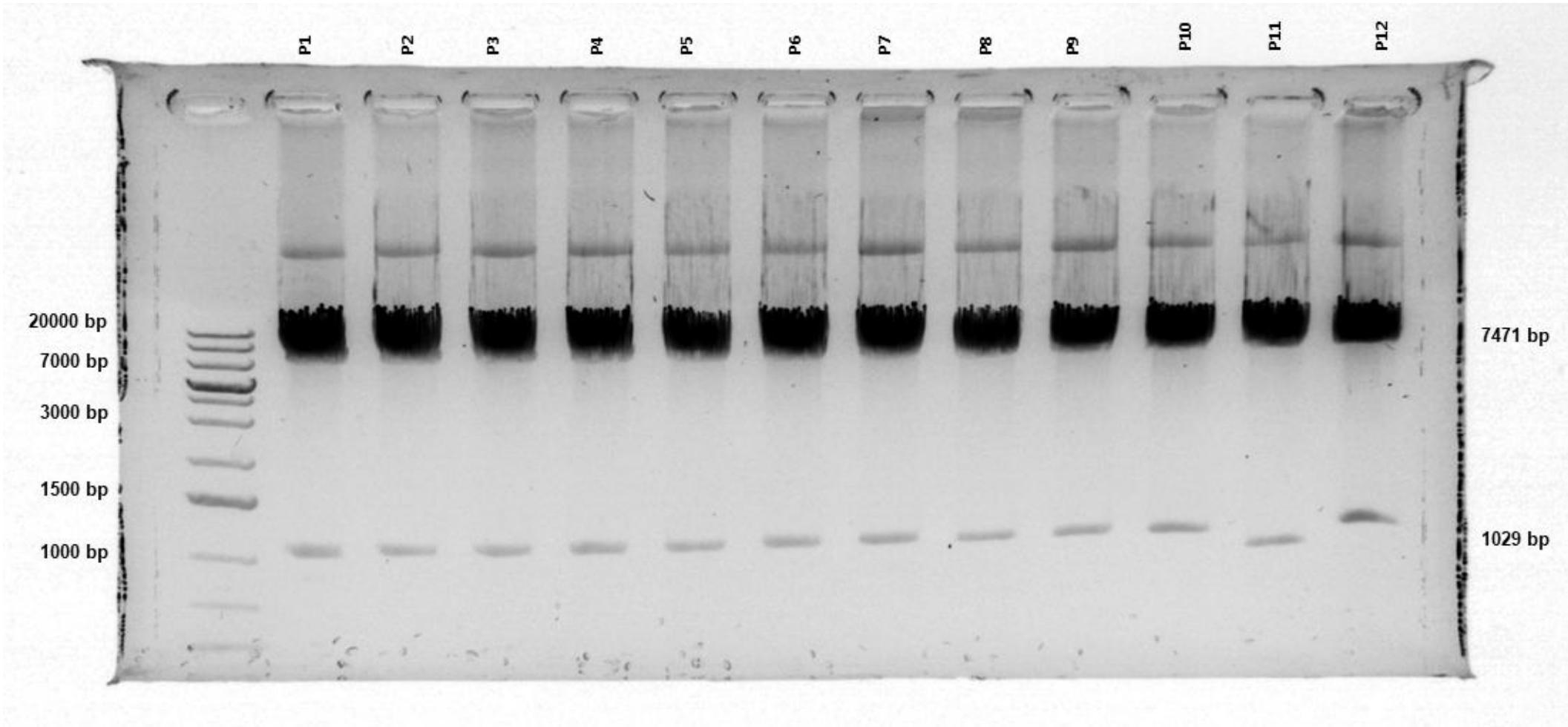

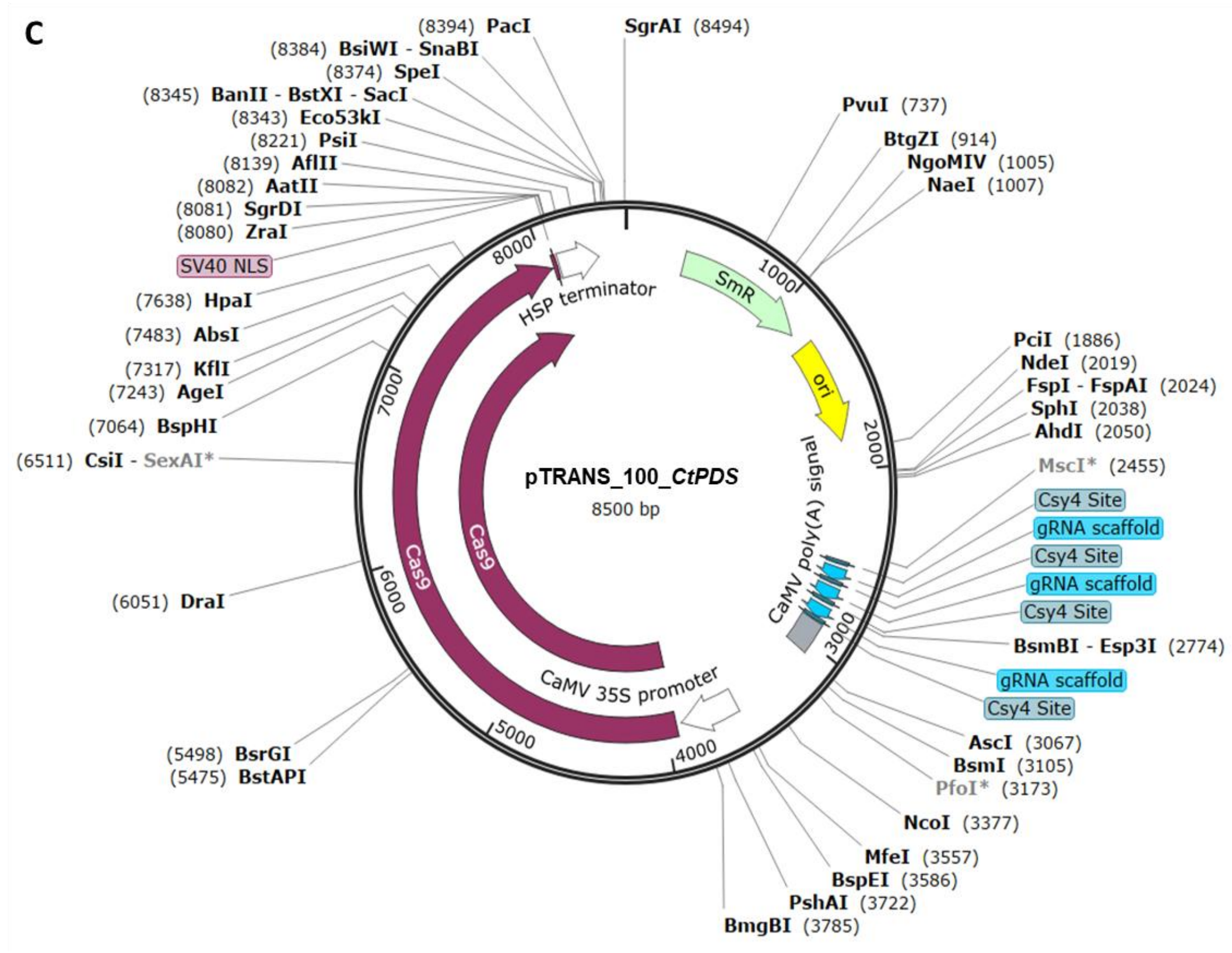

SUPPLEMENTARY FIGURE 3. CRISPR/Cas9 vector constructs developed for *CtPDS* gene editing in guar. (A) Two-step cloning strategy combining Golden Gate assembly and T4 DNA ligation to generate the pTRANS_100_*CtPDS* construct. (B) Gel image of pTRANS_100_*CtPDS* cloning confirmation by restriction digestion with *AscI* and *SphI* enzymes. (C) Final visual representation of the assembled CRISPR/Cas9 non-binary vector (pTRANS_100_*CtPDS*) designed for PEG-mediated protoplast transformation in guar. CaMV35S, cauliflower mosaic virus 35S promoter; CmYLCV, cestrum yellow leaf curling virus promoter; *CtPDS*, *Cyamopsis tetragonoloba phytoene desaturase*; P1-P12, twelve

pTRANS_100_*CtPDS* samples; RE, restriction enzyme; sgRNA, single-guide RNA; 1 kb DNA ladder as size marker.

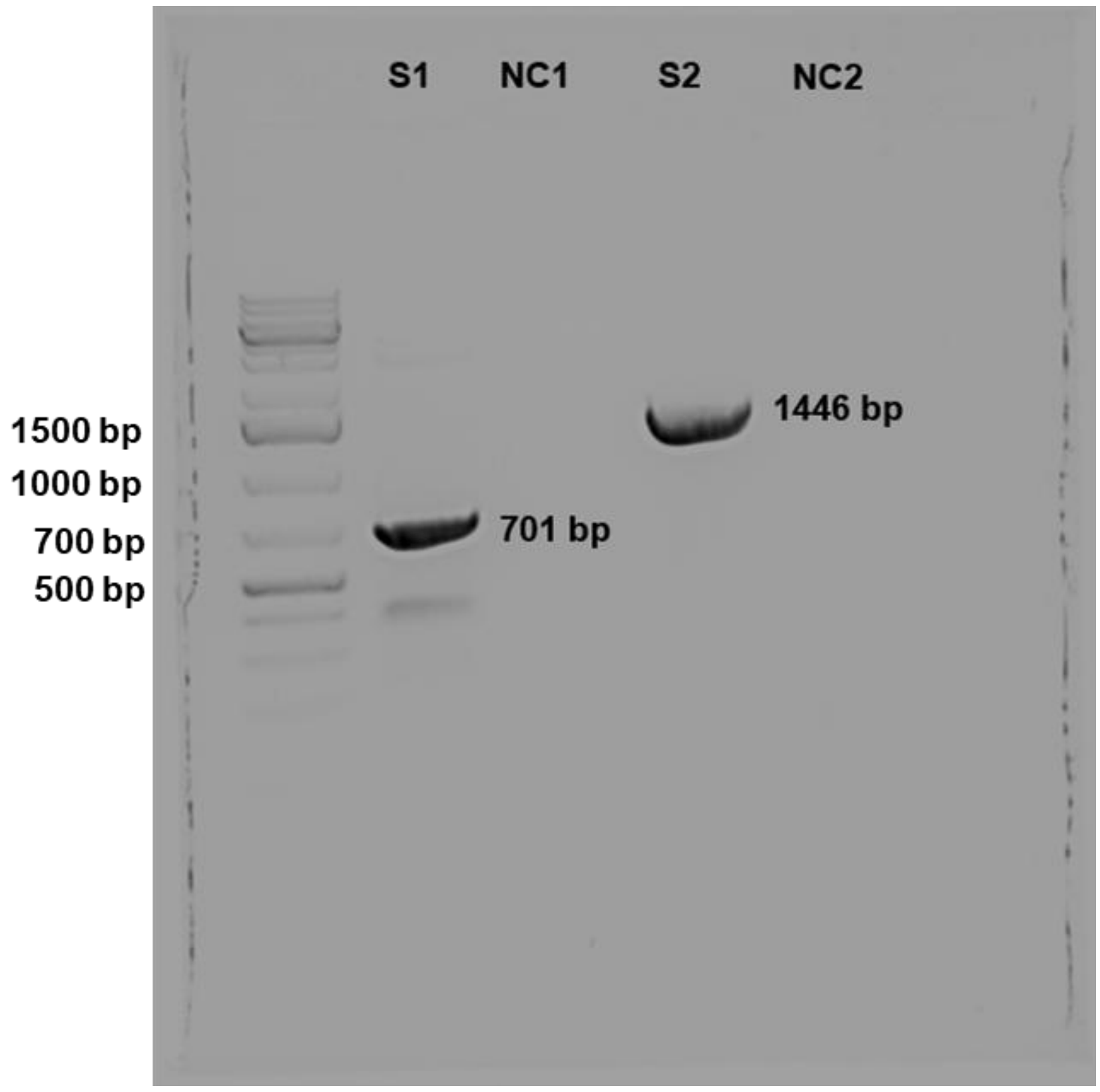

SUPPLEMENTARY FIGURE 4. Validation of *Cyamopsis tetragonoloba phytoene desaturase* gene amplification in guar by PCR. Agarose gel electrophoresis showing PCR products amplified using gene-specific primers. NC, negative control; S1-S2, samples 1 and 2; 1 kb DNA ladder as size marker.

SUPPLEMENTARY TABLE 1. BLAST alignment results of 13 soybean PDS exons against the guar genome, including exon lengths, alignment metrics, sequence identity, coverage, E-value, bit score, and genomic range.

Exon ID	Soybean exon (query) length (bp)	Guar exon (subject) length (bp)	Alignment length (bp)	Matches	Identity (%)	Gaps (%)	Query coverage (%)	Subject coverage (%)	E-value	Bit score	Genomic range (bp)
Exon 1	319	322	337	247	73	9	100	100	6e-53	210	19707622-19707943
Exon 2	89	89	86	80	93	0	97	97	2e-28	126	19707057-19707142
Exon 3	58	56	56	49	88	0	97	100	1e-11	70.7	19706711-19706766
Exon 4	156	156	156	147	94	0	100	100	5e-62	239	19705916-19706071
Exon 5	149	149	149	136	91	0	100	100	2e-53	211	19705607-19705755
Exon 6	120	120	120	116	97	0	100	100	2e-50	200	19705353-19705472
Exon 7	214	214	214	198	93	0	100	100	2e-82	307	19704934-19705147
Exon 8	103	103	103	96	93	0	100	100	4e-36	152	19704478-19704580
Exon 9	48	48	48	46	96	0	100	100	2e-14	78.8	19704046-19704093
Exon 10	46	46	46	43	93	0	100	100	8e-12	70.7	19700832-19700877
Exon 11	188	188	188	172	91	0	100	100	1e-70	268	19700432-19700619
Exon 12	163	163	163	157	96	0	100	100	7e-71	268	19700109-19700271
Exon 13	60	60	60	49	82	0	100	100	3e-08	59.9	19699576-19699635

SUPPLEMENTARY TABLE 2. sgRNAs targeting *CtPDS* exons with their sequences, specificity scores, on-target efficiency, out-of-frame prediction, Lindel score, and off-target information.

Exon no.	sgRNA ID	Target Sequence	MIT Specificity Score	CFD Specificity Score	Doench'16	Moreno-Mateos	Doench-RuleSet3	Out-of-frame	Lindel	Off-target
Exon 1	sgRNA1	GTGCTAAGATCACCAAGTTGAGG	98	99	63	56	73	69	77	0-0-0-0-7
Exon 2	sgRNA2	GAGGCAAGAGATGTTCTAGGTGG	97	99	70	68	95	54	77	0-0-0-2-7
Exon 3	sgRNA3	GATGGAGACTGGTATGAGACGGG	99	100	63	55	37	75	87	0-0-0-1-9

Footnotes

Target Sequence: 20-nucleotide protospacer sequence followed by PAM sequence (underlined). PAM sequences are NGG motifs required for Cas9 recognition and cleavage.

MIT Specificity Score: Predicts off-target binding potential using position-weighted mismatch penalties (0-100 scale, higher is more specific). Calculated using the MIT CRISPR Design Tool algorithm (Hsu et al., 2013).

CFD Specificity Score: Cutting Frequency Determination score predicts off-target cleavage efficiency accounting for mismatch position and identity (0-100 scale, higher is more specific) (Doench et al., 2016).

Doench'16 Score: On-target activity score predicting sgRNA cleavage efficiency using sequence features and chromatin accessibility (0-100 scale, higher is better) (Doench et al., 2016).

Moreno-Mateos Score: On-target efficiency score optimized for prediction in zebrafish and other model organisms (0-100 scale, higher is better) (Moreno-Mateos et al., 2015).

Doench-RuleSet3 Score: Updated on-target activity prediction incorporating additional sequence context and experimental data (0-100 scale, higher is better) (DeWeirdt et al., 2022).

Out-of-frame Score: Probability (percentage) that NHEJ-mediated repair will result in frameshift mutations rather than in-frame deletions (Bae et al., 2014).

Lindel Score: Predicts indel outcome frequencies and frameshift potential at the target site using machine learning (0-100 scale) (Chen et al., 2019).

Off-target Format: Represents potential off-target sites with 0-1-2-3-4 mismatches in the format "n0-n1-n2-n3-n4", where each number indicates the count of genomic sites with the corresponding number of mismatches. For example, "0-0-0-0-7" indicates 0 sites with 0-3 mismatches and 7 sites with 4 mismatches.

References

Bae, S., Kweon, J., Kim, H. S., & Kim, J. S. (2014). Microhomology-based choice of Cas9 nuclease target sites. *Nature Methods*, 11(7), 705-706. https://doi.org/10.1038/nmeth.3015

Chen, W., McKenna, A., Schreiber, J., Haeussler, M., Yin, Y., Agarwal, V., Noble, W. S., & Shendure, J. (2019). Massively parallel profiling and predictive modeling of the outcomes of CRISPR/Cas9-mediated double-strand break repair. *Nucleic Acids Research*, 47(15), 7989-8003. https://doi.org/10.1093/nar/gkz487

DeWeirdt, P. C., McGee, A. V., Zheng, F., Nwolah, I., Hegde, M., & Doench, J. G. (2022). Accounting for small variations in the tracrRNA sequence improves sgRNA activity predictions for CRISPR screening. Nature Communications, 13(1), 5255. https://doi.org/10.1038/s41467-022-33024-2

Doench, J. G., Fusi, N., Sullender, M., Hegde, M., Vaimberg, E. W., Donovan, K. F., Smith, I., Tothova, Z., Wilen, C., Orchard, R., Virgin, H. W., Listgarten, J., & Root, D. E. (2016). Optimized sgRNA design to maximize activity and minimize off-target effects of CRISPR-Cas9. *Nature Biotechnology*, 34(2), 184-191. https://doi.org/10.1038/nbt.3437

Hsu, P. D., Scott, D. A., Weinstein, J. A., Ran, F. A., Konermann, S., Agarwala, V., Li, Y., Fine, E. J., Wu, X., Shalem, O., Cradick, T. J., Marraffini, L. A., Bao, G., & Zhang, F. (2013). DNA targeting specificity of RNA-guided Cas9 nucleases. *Nature Biotechnology*, 31(9), 827-832. https://doi.org/10.1038/nbt.2647

Moreno-Mateos, M. A., Vejnar, C. E., Beaudoin, J. D., Fernandez, J. P., Mis, E. K., Khokha, M. K., & Giraldez, A. J. (2015). CRISPRscan: designing highly efficient sgRNAs for CRISPR-Cas9 targeting in vivo. *Nature Methods*, 12(10), 982-988. https://doi.org/10.1038/nmeth.3543
